# Charge-Separation and Charge-Recombination Rate Constants in a Donor–Acceptor Buckybowl-Based Supramolecular Complex: Multistate and Solvent Effects

**DOI:** 10.1021/acs.jpca.1c05740

**Published:** 2021-11-12

**Authors:** Jesús Cerdá, Joaquín Calbo, Enrique Ortí, Juan Aragó

**Affiliations:** Instituto de Ciencia Molecular (ICMol), Universidad de Valencia, Catedrático José Beltrán 2, Paterna 46980, Spain

## Abstract

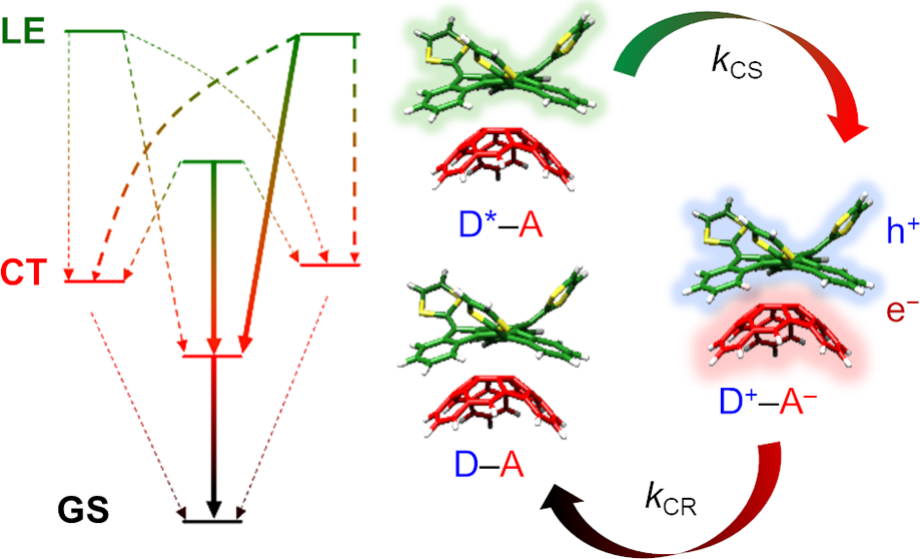

The kinetics of the nonradiative photoinduced processes (charge-separation and charge-recombination) experimented in solution by a supramolecular complex formed by an electron-donating bowl-shaped truxene-tetrathiafulvalene (truxTTF) derivative and an electron-accepting fullerene fragment (hemifullerene, C_30_H_12_) has been theoretically investigated. The truxTTF·C_30_H_12_ heterodimer shows a complex decay mechanism after photoexcitation with the participation of several low-lying excited states of different nature (local and charge-transfer excitations) all close in energy. In this scenario, the absolute rate constants for all of the plausible charge-separation (CS) and charge-recombination (CR) channels have been successfully estimated using the Marcus–Levich–Jortner (MLJ) rate expression, electronic structure calculations, and a multistate diabatization method. The outcomes suggest that for a reasonable estimate of the CS and CR rate constants, it is necessary to include the following: (i) optimally tuned long-range (LC) corrected density functionals, to predict a correct energy ordering of the low-lying excited states; (ii) multistate effects, to account for the electronic couplings; and (iii) environmental solvent effects, to provide a proper stabilization of the charge-transfer excited states and accurate external reorganization energies. The predicted rate constants have been incorporated in a simple but insightful kinetic model that allows estimating global CS and CR rate constants in line with the most generalized three-state model used for the CS and CR processes. The values computed for the global CS and CR rates of the donor–acceptor truxTTF·C_30_H_12_ supramolecular complex are found to be in good agreement with the experimental values.

## Introduction

Since their discovery,^1,2^ organic solar cells (OSCs) have been considered as potential alternatives to silicon photovoltaic cells, mainly due to their low cost, easy processing, and low toxicity.^3^ Although there has been no improvement in the performance of OSCs for many years, recent breakthroughs pushing the performance above 17% have again reawakened the interest in this photovoltaic technology.^4,5^ OSCs are usually made of an active layer formed by a mixture of organic semiconducting molecules with donor and acceptor characteristics (bulk heterojunction), which is sandwiched between two electrodes. In general, the processes occurring in a standard OSC can be summarized as follows: (i) light absorption by the donor compound (exciton formation), (ii) exciton migration to the interface between the donor and acceptor, and (iii) electron transfer from the donor to the acceptor (*i.e*., charge separation, CS), with the consequent generation of a charge-transfer (CT) state. At this point, two possible paths can take place, either (iv) the detrimental process by which the separated charges recombine coming back to the ground state (*i.e*., charge recombination, CR) or (v) the generated charges overcome the Coulombic attraction and migrate to the respective electrodes giving rise to the desired photocurrent.

**Figure 1:**
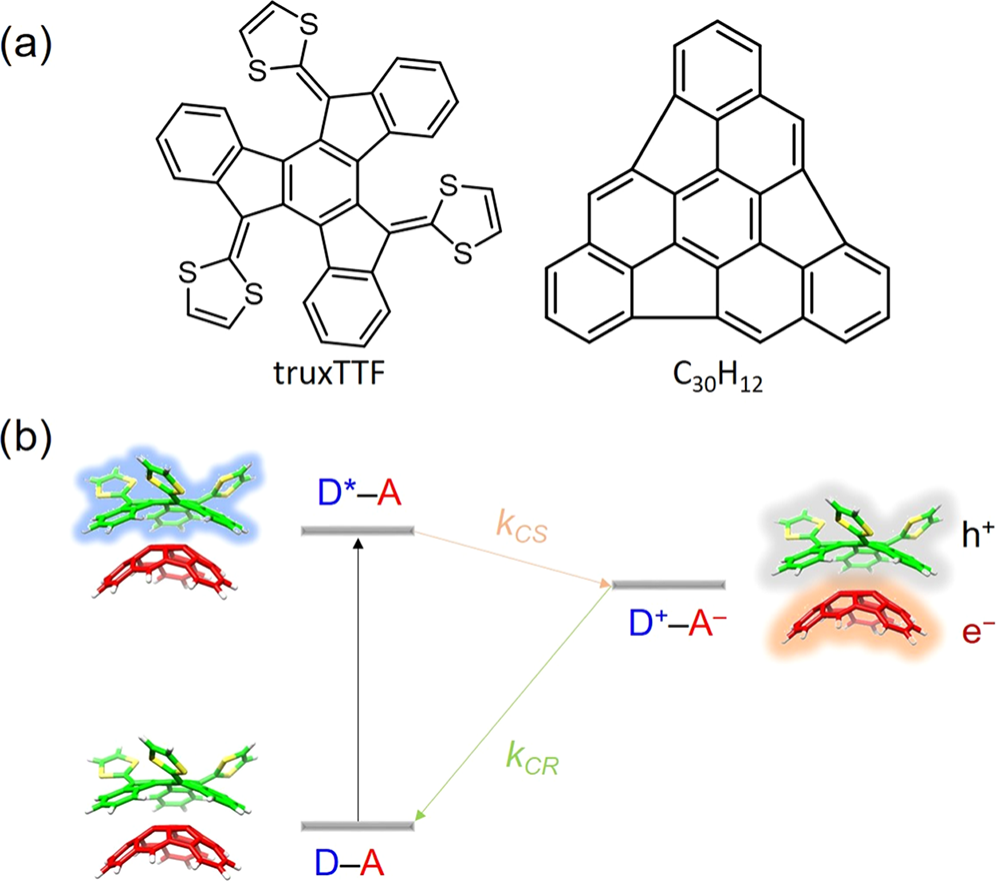
(a) Chemical structures of the donor truxTFF and the acceptor buckybowl C_30_H_12_ (hemifullerene). (b) Schematic representation of the charge-separation and charge-recombination photophysical processes taking place in the donor–acceptor supramolecular truxTTF·C_30_H_12_ complex.

If we turn our attention to the active materials involved in OSCs, fullerenes and fullerene derivatives are the most used electron-acceptor systems for OSC applications.^6−9^ In particular, [6,6]-phenyl-C_61_-butyric acid methyl ester (known as PCBM) is likely to be the most employed acceptor for bulk heterojunction solar cells.^10−12^ The combination of PCBM with poly(3-hexylthiophene) (P3HT), acting as a donor, has been widely studied as a model system to gain insight into the elementary physical processes occurring in OSCs.^3,13−15^ In the last years, the quest for novel non-fullerene acceptors for photovoltaic applications has emerged as an active research field to boost the potential and application of OSCs.^16−19^ Recently, novel fullerene fragments known as buckybowls (*e.g*., C_30_H_12_, C_32_H_12_, and C_38_H_14_) have been synthesized (Figure 1).^20−22^ These buckybowls mimic the electron-acceptor behavior of C_60_ when combined supramolecularly with the truxene-tetrathiafulvalene (truxTTF) electron-donor derivative (Figure 1), exhibiting an efficient photoinduced CS process and a slower CR event, and may therefore be considered as potential candidates in the context of OSCs.^23,24^

From a computational perspective, the usual approximations to evaluate the CS and CR rate constants rely on the use of the classical Marcus equation or the semiclassical Marcus–Levich–Jortner (MLJ) variant.^25,26^ The use of these two rate expressions requires the accurate estimation of the energies of the initial and final electronic states involved in the electron-transfer process. Time-dependent density functional theory (TDDFT) is likely to be the electronic structure method most widely used to theoretically estimate the energy position of the excited states implied in photoinduced electron-transfer events at a molecular scale. Nevertheless, TDDFT, when combined with standard GGA and hybrid density functionals, holds inherent drawbacks concerning the energy prediction of CT excited states (usually a significant underestimation) due to self-interaction errors.^27−29^ Long-range corrected (LC) density functionals (*e.g*., LC-ωPBE,^27^ CAM-B3LYP,^30^ or ωB97X-D,^31^ just to mention a few) were specially designed to mitigate this general drawback and other weaknesses. Although standard LC density functionals can be generally employed for different chemical applications,^32^ optimally tuned LC density functionals have been demonstrated to behave more accurately for electron-transfer problems in donor–acceptor molecular heterojunctions.^33−37^ In particular, optimally tuned LC density functionals are able to provide a balanced description of both local and CT excitations due to the system-dependent tuning based on physical grounds.

Solvent effects play an essential role in stabilizing the CT states and, therefore, in determining the relative energy of the excited states when the electron-transfer reaction occurs in solution. The electron-transfer rate expressions indeed require the estimation of the Gibbs free energy difference between fully relaxed states, which, in addition to geometric relaxation, implies solvent polarization and relaxation. In this context, the widely employed polarization continuum model (PCM)^38^ in its standard linear-response TDDFT formalism cannot be totally adequate to capture the stabilization of the excited CT states since the solvent response acts only on the transition densities of the targeted CT states. An alternative state-specific PCM formulation,^39^ where solvent–solute interactions are evaluated through the specific density of the excited states of interest, has been successfully developed to accurately capture solvent effects for spectroscopic purposes (simulation of the emission spectra and Stokes shifts).^40^ Nonetheless, this state-specific PCM method has been barely employed in charge-transfer contexts.^41^ In addition to continuum models, polarizable force fields explicitly including solvent molecules surrounding the supramolecular complexes have been employed to describe CT excited states in solution.^42^

Another critical aspect in the evaluation of nonadiabatic photoinduced electron-transfer rates is the estimation of the electronic couplings between the excited states involved in the electron-transfer processes.^43^ Electronic couplings are usually computed using either the orbital interaction model^44,45^ or two-state diabatization schemes (*e.g*., the generalized Mulliken–Hush^46^ or the fragment charge difference (FCD)^47^ treatments). Both approaches present drawbacks; the former fails when more than a single monoexcitation is needed for the correct excited state description, whereas the latter may provide wrong electronic-coupling values when several excited states close in energy (*i.e*., multistate effects) are involved in the electron-transfer processes. The relevance of multistate effects on the electronic couplings was already discussed by Cave et al.^48^ in small-size molecular pairs but, surprisingly, has been hardly discussed in the context of OSCs for donor–acceptor heterojunction models. Recently, Kastinen et al.^49^ have stressed its importance for poly(thiophene-*co*-quinoxaline)–PC_71_BM interfaces.

In the present work, we propose a theoretical protocol to accurately estimate the charge-separation and charge-recombination rates in donor–acceptor (D–A) supramolecular assemblies in solution. As a model system, we have selected a D–A supramolecular heterodimer formed by the electron-donor truxTTF and the hemifullerene C_30_H_12_ as the electron acceptor (Figure 1). The theoretical approximation here presented combines the Marcus–Levich–Jortner rate expression^50−52^ and DFT electronic structure calculations, along with TDDFT excited-state energy levels, to carefully evaluate the different terms entering the rate expression, *i.e*., Gibbs free energy differences, electronic couplings, and reorganization energies. For D–A supramolecular complexes involving fullerene fragments (in particular truxTTF·C_30_H_12_), and by extension fullerenes, a complex scenario with a set of low-lying, close-in-energy excited states of local and CT character is generally found, opening the door to multiple CS and CR pathways. We demonstrate that in this situation, the inclusion of multistate effects in the selected diabatization scheme is mandatory to predict reasonable electronic couplings for the different electron-transfer channels. We also highlight the relevance of the theoretical solvent model to adequately capture the energy stabilization of the CT excited states due to solvent effects (polarization and reorganization).

## Methodology

### Rate Constant Expression

Assuming a weak electronic-coupling regime, the photoinduced electron-transfer events in buckybowl-based donor–acceptor supramolecular complexes can be described within a hopping mechanism by a nonadiabatic electron-transfer rate expression. Among the different electron-transfer rate expressions, the Marcus–Levich–Jortner equation was selected because it is able to incorporate quantum tunneling effects. Note that in π-conjugated semiconducting compounds, high-frequency vibrations (associated with single and double carbon–carbon stretching motions) significantly couple to the electronic states responsible for the electron-transfer processes. In contrast to the classical Marcus theory, the MLJ rate expression is able to capture quantum effects that come from the high-frequency vibrations through an effective vibrational normal mode coordinate.^50−54^ The MLJ nonadiabatic electron-transfer rate is expressed as follows
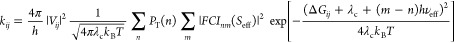
1where *V*_*ij*_ is the electronic coupling between the initial *i* and final *j* electronic states, *k*_B_ is the Boltzmann constant, *T* is the temperature, *h* is the Planck constant, and Δ*G*_*ij*_ is the Gibbs free energy difference between the initial and final states. *λ*_c_ corresponds to the classical reorganization energy including intramolecular and external (solvent) components (*vide infra*). *FCI*_*nm*_ (S_eff_) denotes the Franck–Condon integral between the initial (*n*) and final (*m*) vibrational levels of the initial *i* and final *j* electronic states, which are calculated using an analytic expression under the harmonic approximation (see eq S1 in the Supporting Information).^55^ The Franck–Condon integral depends on the Huang–Rhys (HR) factor *S*_eff_, which describes the relative displacement along an effective quantum normal mode with frequency *ν*_eff_. Finally, *P*_T_(*n*) represents the Boltzmann probability that a vibrational state *n* on an initial electronic state *i* is occupied at a certain temperature. It should be noted that the rate constant in its current form is only valid for electron-transfer events within the limiting incoherent regime, where the involved electronic states in the diabatic picture are localized at molecular units. For delocalized situations, a more general rate expression would be necessary (see ref (56)).

### Electronic Couplings

In a system with *N* adiabatic (AD) electronic states {ψ_1_, ψ_2_, ..., ψ_*N*_} with energies {*E*_1_, *E*_2_, ..., *E*_*N*_}, the adiabatic Hamiltonian matrix is diagonal (**H**^**AD**^). These AD states can be related to a set of *N* diabatic (DI) states {φ_1_, φ_2_, ..., φ_*N*_} by means of an orthogonal transformation as follows
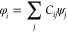
2

Once the adiabatic-to-diabatic orthogonal transformation matrix **C** is determined, the diabatic and adiabatic Hamiltonians are easily connected by **H**^**DI**^**= CH**^**AD**^**C**^**T**^ (diabatization), where the diagonal elements of **H**^**DI**^ correspond to the diabatic energies and the off-diagonal elements to the electronic couplings (*V*_*ij*_). Although there is no unique adiabatic-to-diabatic transformation,^57^ most diabatization schemes, particularly in the context of charge/energy transfer, aim to find the best unitary transformation matrix **C** that generates the closest diabatic states with respect to a set of reference states with a well-defined molecular property. Among the most popular diabatization schemes for charge transfer, the generalized Mulliken–Hush method, which employs (transition) dipole moments,^46^ and the fragment charge difference (FCD) scheme,^47^ which uses a charge difference operator, have to be emphasized. In this study, the FCD diabatization scheme within its multistate extension^58^ was selected (see the Supporting Information for a brief description of the FCD method). We anticipate that the inclusion of multistate effects is crucial for accurate electronic-coupling predictions in D–A truxTTF·C_30_H_12_ owing to the presence of a number of low-lying singlet excited states in a narrow energy range.

### Singlet Excited States and Gibbs Free Energy Difference

To describe appropriately the CS and CR processes in the D–A interface at the molecular level, it is necessary to characterize the lowest-energy singlet excited states of the supramolecular heterodimer model (in our case, truxTTF·C_30_H_12_). Triplet excited states are not considered in this work, although they may also play an active role in the photoinduced electron-transfer events in bulk heterojunctions.^59−62^ The low-lying singlet excited states of the truxTTF·C_30_H_12_ complex were computed within the TDDFT approach in its Tamm–Dancoff (TDA–DFT) variant. The lowest-energy excited states in the D–A truxTTF·C_30_H_12_ heterodimer were expected to be relatively close in energy and show different nature (*i.e*., local excitations (LE) centered in the donor/acceptor fragments or charge-transfer (CT) excitations). It is well-known that the energy estimation of CT excited states is a challenging task for TDDFT with GGA and hybrid density functionals due to self-interaction errors.^27−29,63^ In contrast, long-range corrected (LC) density functionals have been demonstrated to provide a satisfactory description of CT-like excited states,^63,64^ especially when these LC functionals have been optimally tuned (OT) for the specific system under study.^36,63,65−67^ The tuning procedure used here was carried out in the gas phase according to eq S4(37,65,67) and was performed for both the isolated compounds (truxTTF and C_30_H_12_) and the supramolecular truxTTF·C_30_H_12_ assembly (see the Supporting Information for further details).

The adiabatic Gibbs free energy difference for CS and CR processes was computed as follows
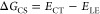
3
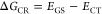
4Assuming that the entropic component is negligible, Δ*G*_CS/CR_ can be approximated as the electronic energy difference (Δ*E*) between the involved states at their respective minimum-energy geometry. *E*_CT_ denotes the energy of a D^+^–A^–^ CT-like excited state (truxTTF^+^·C_30_H_12_^–^), whereas *E*_LE_ corresponds to the energy of a local-type excited state where the excitation is mainly localized on the donor (*i.e*., truxTTF*·C_30_H_12_). *E*_GS_ denotes the ground-state energy of the truxTTF·C_30_H_12_ heterodimer.

### Reorganization Energy

The reorganization energy (λ) is a key parameter for the evaluation of electron-transfer rates and is associated with the energy change owing to electronic redistribution and nuclear rearrangement in the electron-transfer events.^68^ Generally, λ is split into internal and external reorganization components (λ = λ_int_ + λ_ext_). The former accounts for the energy cost of the intramolecular nuclear relaxation of the donor and acceptor systems associated with the electron-transfer reaction. The latter comes from the environmental effects resulting from the polarization and reorientation of neighboring molecules as a response to the charge (electron or hole) injection in the donor–acceptor system.

The internal reorganization energies for the CS and CR processes (λ_int_^CS^ and λ_int_^CR^) are expected to be different. For the electron-transfer D*–A → D^+^–A^–^ reaction (CS), λ_int_^CS^ corresponds to the energy difference between the initial geometry (D*–A) and the final geometry (D^+^–A^–^) in the potential energy surface of the CT D^+^–A^–^ excited state. For the CR process (D^+^–A^–^ → D–A), λ_int_^CR^ is estimated as the energy change between the initial geometry (D^+^–A^–^) and the final geometry (D–A) in the ground-state potential energy surface. Internal reorganization energies can be decomposed into contributions for each vibrational normal mode according to λ_int_^CS/CR^ = ∑_*k*_*h*ν_*k*_*S*_*k*_,^52^ where ν_*k*_ is the vibrational frequency of the normal mode *k* and *S*_*k*_ denotes the corresponding HR factor.^69^ The electron-transfer reaction can be drastically but successfully simplified^70−72^ to a single effective normal mode coordinate with frequency *ν*_eff_ = ∑_*k*_ν_*k*_*S*_*k*_ /∑_*k*_*S*_*k*_ and an effective HR factor *S*_eff_ = λ_int_^CS/CR^/*hν*_eff_.^44^

Concerning the external reorganization energy, there are several methods to estimate λ_ext_ in solution with different degrees of accuracy. Among them, the Marcus two-sphere model,^73^ the “nonequilibrium versus equilibrium solvation” approximation,^39^ and the dynamic polarization response method should be stressed.^74^ Note that the calculation of λ_ext_ in solution is always associated with a significant uncertainty, and possibly, it is the parameter subject to a larger error in the electron-transfer rate expressions. In molecular crystals, λ_ext_ can be successfully evaluated based on hybrid techniques combining electronic structure calculations and polarizable force fields.^75^ Nevertheless, λ_ext_ is generally small compared to λ_int_ and, therefore, less determining for the charge-transfer rates in molecular crystals. In the present work, the “nonequilibrium versus equilibrium solvation” approximation within the state-specific polarizable continuum model (SS-PCM)^39^ is employed. The SS-PCM method has been already proved to satisfactorily estimate the inhomogeneous broadening of electronic transitions in solution^76^ and to capture solvent effects in electron-transfer reactions.^41^ Briefly, the method allows using an initial solvent configuration (nonequilibrium), where only fast polarization effects are captured, and a final solvent configuration (equilibrium), where the slow solvent reorientation has occurred. The difference between these two situations (*E*_Eq_ – *E*_NonEq_) gives an estimate of the total λ (λ = λ_int_ + λ_ext_).

### Computational Details

All of the calculations were performed using the Gaussian16 package in its revision A03,^77^ except for the ground-state geometries of the truxTTF·C_30_H_12_ supramolecular complex, which were extracted from previously published results at the revPBE0-D3/cc-pVTZ level.^23^ Singlet excited-state calculations were performed within the TDDFT approach in its TDA–DFT variant^78^ using different density functionals in combination with the Pople’s 6-31G** basis set.^79^ The GGA BLYP^80,81^ and hybrid B3LYP^80,81^ functionals, as well as the long-range corrected density functionals LC-BLYP,^64^ CAM-B3LYP,^30^ LC-ωPBE,^27^ and ωB97X-D^82^ according to the Hirao’s correction,^64^ were employed. Likewise, the optimally tuned versions of LC-BLYP, LC-ωPBE, and ωB97X-D (hereafter named OT-LC-BLYP, OT-LC-ωPBE, and OT-ωB97X-D, respectively) were also used. Solvent effects were taken into account within the polarizable continuum model (PCM)^74,83^ with *o*-dichlorobenzene as the solvent. In the case of CT excited states, their energies were recalculated by performing single-point calculations using the SS-PCM approach^39^ and the linear-response PCM-optimized geometries, to properly account for the CT stabilization due to environmental polarization and reorganization effects.

Electronic couplings *V*_*ij*_ were estimated using the standard two-state FCD diabatization scheme^43^ and a multistate extension^58^ implemented in a home-made program. The program makes use of the overlap matrix between the atomic basis functions, the molecular orbital coefficients, and the excitation coefficients obtained from TDA–DFT calculations.

## Results and Discussion

### Supramolecular Heterodimer Structures

**Figure 2:**
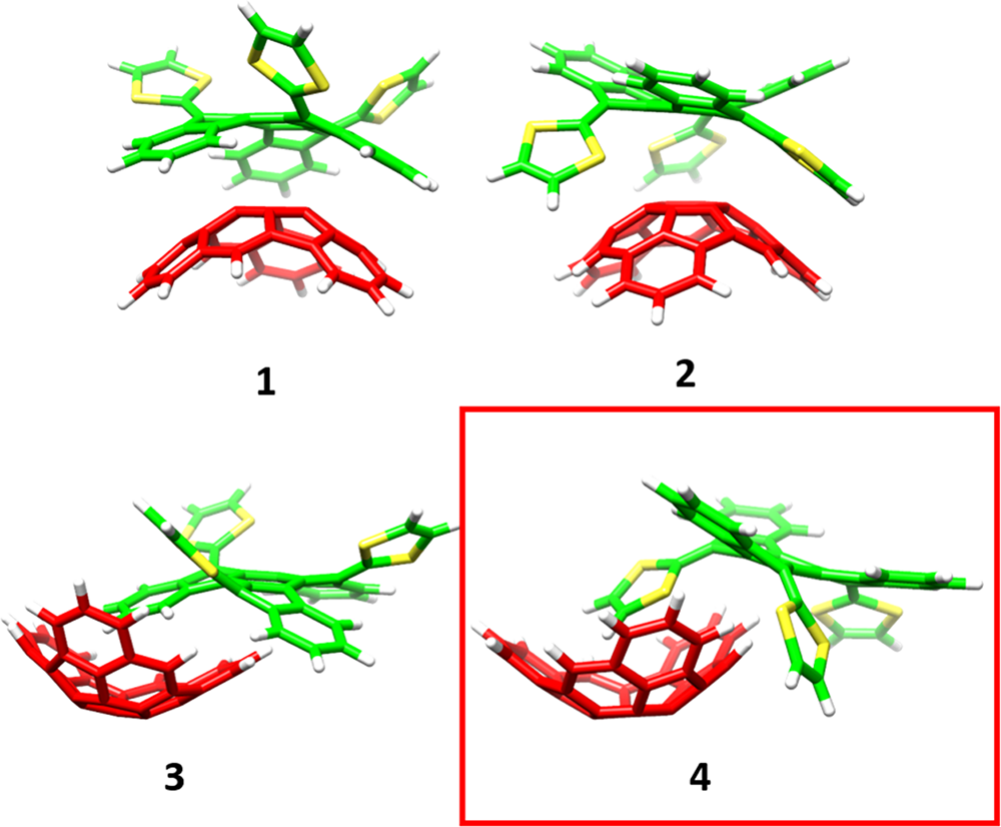
Minimum-energy structures computed at the revPBE0-D3/cc-pVTZ level of theory^23^ for the truxTTF·C_30_H_12_ supramolecular donor–acceptor heterodimer.^23^ For truxTTF: carbon atoms in green, sulfur in yellow, and hydrogen in white. For C_30_H_12_: carbon in red and hydrogen in white. The most stable structure **4** (mainly used during the discussion) is highlighted with a red square.

Four different minimum-energy structures of the supramolecular truxTTF·C_30_H_12_ heterodimer were previously reported, as shown in Figure 2.^23^ The structures were calculated at the revPBE0-D3/cc-pVTZ level and exhibited close intermolecular contacts in the 2.5–4.0 Å range, indicative of favorable noncovalent interactions between the electron-donor truxTTF and the electron-acceptor C_30_H_12_ bowl. In structures **1** and **2** (bowl-in-bowl structures), the convex surface of the C_30_H_12_ bowl matches the two concave cavities of the truxTTF bowl; in structure **1**, the C_30_H_12_ bowl interacts with the carbon backbone of truxTTF, whereas in structure **2**, the bowl faces the cavity formed by the central benzene and the three dithiole rings. In structures **3** and **4** (staggered structures), the truxTTF is placed inside the C_30_H_12_ cavity; in structure **3**, a benzene ring is in the cavity, whereas in structure **4**, a dithiol ring is inside of C_30_H_12_. Staggered structures were predicted to be more stable than the bowl-in-bowl conformers due to the larger number of CH−π and π–π interactions that take place in the former. In particular, interaction energies of −25.3 and −28.1 kcal mol^–1^ were computed for structures **3** and **4**, respectively, compared with the values of −21.0 and −19.4 kcal mol^–1^ obtained for structures **1** and **2**, respectively.^23^ From now on, we will keep our discussion focused on the most stable structure **4**, as this staggered structure is likely to be the most abundant in solution.^24^ Nonetheless, analysis of the CS and CR rate constants and relevant parameters calculated for structures **1**–**3** are given in the Supporting Information.

### Analysis of the Low-Lying Singlet Excited States

Prior to calculating Δ*G*_CS_ and Δ*G*_CR_, which requires excited-state geometry optimizations, it is desirable to perform an analysis of the excited-state distribution at the ground-state geometry (Franck–Condon region) of truxTTF·C_30_H_12_ (structure **4**). Density functionals of different nature—GGA (BLYP), hybrid (B3LYP), and CAM-B3LYP, together with the optimally tuned long-range corrected functionals OT-LC-BLYP, OT-LC-ωPBE, and OT-ωB97X-D—were initially evaluated within the TDA–DFT approximation (6-31G** basis set in *o*-dichlorobenzene), to investigate and assess their performance. Among the analyzed density functionals, the OT-LC-BLYP(ω = 0.16 bohr^–1^) functional showed the best performance and, therefore, was adopted for the calculation of the parameters related to the estimation of the CS and CR rate constants (see Section S2 in the Supporting Information for full details).

**Figure 3:**
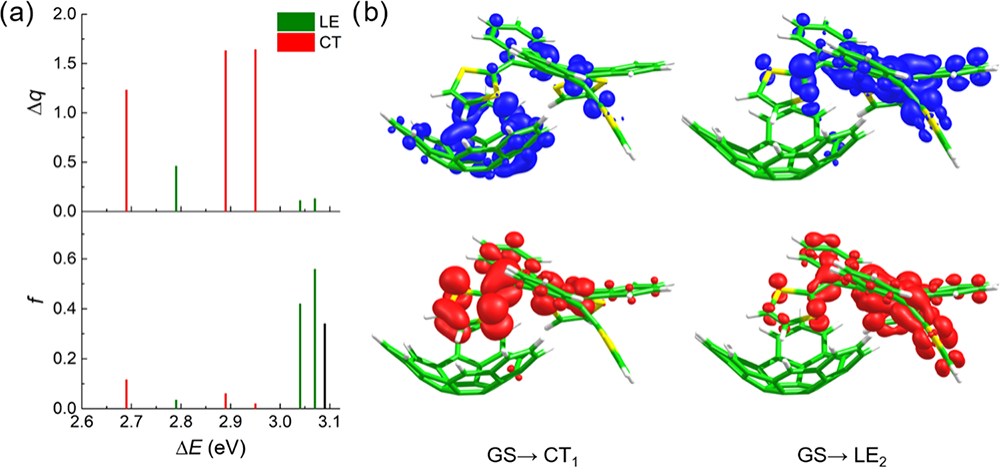
(a) Representation of Δ*q* (top) and oscillator strength (*f*, bottom) as a function of the excitation energy (Δ*E*) calculated for the truxTTF·C_30_H_12_ heterodimer at the OT-LC-BLYP(ω = 0.16 bohr^–1^)/6-31G** level in *o*-dichlorobenzene within the PCM approach. The bright lowest-energy excited states (S_2_ and S_3_) computed for truxTTF at 3.09 eV (bottom, black bar) at the same level of theory are also indicated for comparison purposes. (b) Attachment (top) and detachment (bottom) densities computed for the lowest-energy S_0_ → S_1_ transition of CT nature (GS → CT_1_) and the bright S_0_ → S_5_ transition with LE character (GS → LE_2_).

Figure 3a displays the vertical excitation energies, the oscillator strengths (*f*), and the values of the charge difference between the donor and the acceptor (Δ*q*) calculated for the six lowest-energy singlet excited states of the truxTTF·C_30_H_12_ assembly at the OT-LC-BLYP(ω = 0.16 bohr^–1^)/6-31G** level in the presence of *o*-dichlorobenzene within the PCM approach (see also Table S8). Vertical excitation energies and oscillator strengths for the isolated truxTTF donor are also included for comparison purposes. Δ*q* is used as a descriptor that measures the CT character of a particular excited state (see Section S1.2 in the Supporting Information). States with Δ*q* values above 1e indicate a significant CT character, whereas states with Δ*q* < 0.5e are characteristic of LE excitations involving only the truxTTF donor. Values of Δ*q* between 0.5 and 1e correspond to states with a mixed LE&CT character. All of the electronic transitions relevant for the CS and CR processes in truxTTF·C_30_H_12_ are found to be in the 2.69–3.10 eV energy window and can be classified as LE or CT excitations according to the Δ*q* descriptor^84^ and the attachment/detachment densities (Figure 3b and Table S8). The three lowest-energy CT electronic transitions (S_0_ → S_1_, S_0_ → S_3_, and S_0_ → S_4_, from now on labeled as GS → CT_1_, GS → CT_2_, and GS → CT_3_) are calculated at 2.69, 2.88, and 2.95 eV, respectively, and are mainly described by monoexcitations from the highest occupied molecular orbital (HOMO) of truxTTF to the lowest unoccupied molecular orbitals (LUMOs, LUMO + 1, and LUMO + 2, respectively) of C_30_H_12_ (Figure S3), thus implying a significant charge transfer from the donor to the acceptor. The CT character of these transitions is corroborated by the Δ*q* descriptor with values of 1.23, 1.63, and 1.64e for CT_1_, CT_2_, and CT_3_, respectively (Figure 3a and Table S8), and visualized by the attachment/detachment densities calculated for the GS → CT_1_ (Figure 3b) and GS → CT_2_, CT_3_ transitions (Figure S4).

The S_0_ → S_2_, S_0_ → S_5_, and S_0_ → S_6_ electronic transitions are computed at 2.79, 3.04, and 3.07 eV, respectively, and present small Δ*q* values (0.46, 0.11, and 0.13, respectively), indicative of their LE character, as supported by the attachment/detachment densities calculated for the S_0_ → S_5_ (Figure 3b). These transitions are hereafter named GS → LE_1_, GS → LE_2_, and GS → LE_3_, respectively. While the GS → LE_1_ transition exhibits a small oscillator strength (*f* = 0.035), the GS → LE_2_ and GS → LE_3_ excitations correspond to bright transitions with *f* values of 0.420 and 0.558, respectively, in line with the electronic transitions calculated for isolated truxTTF (Table S11).

The above outcomes, with the presence of at least six singlet excited states close in energy in less than 0.4 eV at the Franck–Condon region, clearly highlight a complex scenario where several charge-transfer channels can occur during the CS and CR electronic events.

### Electronic Couplings

All of the electronic couplings (*V*_*ij*_) between the low-lying LE (LE_1_, LE_2_, and LE_3_) and CT (CT_1_, CT_2_, and CT_3_) excited states and also the ground state for the truxTTF·C_30_H_12_ heterodimer were evaluated within the TDA–DFT approximation at the OT-LC-BLYP(ω = 0.16 bohr^–1^)/6–31G** + PCM (*o*-dichlorobenzene) level using the ground-state geometry and the FCD diabatization scheme^47^ in its two-state and multistate variants (Table 1). The *V*_*ij*_ couplings estimated using the multistate FCD method are found in the 4–44 meV range and are comparable to the values reported in the literature for different D–A supramolecular heterojunctions.^44,49^ Regarding the CS process, the *V*_*ij*_ couplings computed for the electron transfer between the local excited states LE_1–3_ to the first CT_1_ excited state show, in general, the largest values (43.6, 4.1, and 32.8 meV for *V*_LE1–CT1_, *V*_LE2–CT1_, and *V*_LE3–CT1_, respectively). These *V*_*ij*_ values are consistent with the nature of the S_1_ state, which is a CT-like state but with a non-negligible mixing of LE excitations as suggested by the value of Δ*q* (1.23) and the attachment/detachment densities (Figure 3b). For the CR process, the couplings between the three lowest CT_1–3_ states and the ground state are calculated to be 10.0, 0.7, and 4.4 meV, respectively (Table 1). Thus, the CR event between CT_1_ and GS is the most plausible recombination pathway.

**Table 1:** Absolute Electronic Couplings *V*_*ij*_ between the Ground State (GS) and Charge-Transfer (CT) Excited States and between Local (LE) and CT Excited States Calculated Using the FCD Diabatization Scheme in Its Multistate Variant^a^

*V*_*ij*_ (meV)			
	CT_1_	CT_2_	CT_3_
GS	10.0 (270.0)	0.7 (67.1)	4.4 (4.3)
LE_1_	43.6 (43.9)	20.2 (9.6)	14.8 (7.2)
LE_2_	4.1 (10.1)	6.1 (11.2)	9.2 (9.7)
LE_3_	32.8 (97.8)	24.0 (25.0)	9.4 (16.1)

a *V*_*ij*_ values estimated under the two-state FCD approximation are included within parentheses for comparison purposes.

It is interesting to compare the above results with the *V*_*ij*_ couplings calculated by employing the two-state FCD variant, which is the most widely used approach in the electron-transfer context.^47,85^ However, in complex scenarios where a dense manifold of low-lying excited states close in energy is present, as is the case of truxTTF·C_30_H_12_ and many other heterojunction systems,^49^ the two-state FCD approximation is insufficient to provide an adequate description of the electronic couplings between the states involved in the electron-transfer processes. As can be seen in Table 1, the two-state FCD diabatization scheme yields reasonable *V*_*ij*_ couplings for the CS process, with values similar to those obtained with the more accurate multistate FCD variant. An exception is found for the coupling between LE_3_ and CT_1_, with a significantly larger *V*_LE3–CT1_ value of 97.8 meV for the two-state FCD approach compared to the multistate variant (32.8 meV). The large *V*_LE3–CT1_ coupling obtained from the two-state FCD approximation would suggest the electron transfer from the highest-energy local excited state (S_6_) to the lowest CT state (S_1_) as the most probable CS pathway, which is at odds with the multistate FCD picture. Concerning the CR process, the descriptions provided by two-state and multistate FCD approximations are largely divergent. In particular, the *V*_CT1–GS_ and *V*_CT2–GS_ couplings computed with the two-state FCD variant are significantly overestimated (270.0 and 67.1 meV, respectively) compared to the values predicted with the multistate FCD scheme (10.0 and 0.7 meV, respectively). The highly overestimated *V*_CT1–GS_ and *V*_CT2–GS_ values obtained within the two-state FCD scheme are clearly artifacts, highlighting the importance of including multistate effects for accurate coupling predictions in the donor–acceptor truxTTF·C_30_H_12_ heterodimer. The overestimation of the CR couplings by the two-state FCD approach is in line with recent findings reported by Kastinen et al.,^49^ who also employed an optimally tuned LC density functional. Our results therefore reveal that the multistate FCD variant is highly recommended in those cases where different electron-transfer pathways (either charge-separation or charge-recombination) can occur as a consequence of the presence of a large number of excited states in a narrow energy window.

### Gibbs Free Energy Difference

**Figure 4:**
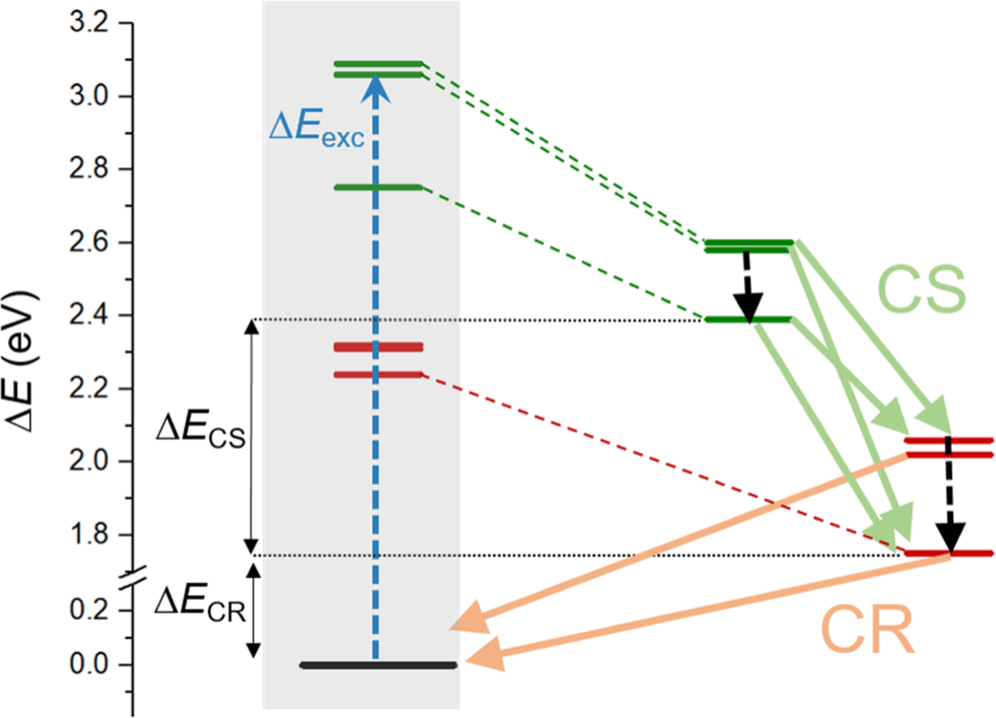
Schematic diagram of the computed excited states of interest indicating the Δ*E*_CS_ and Δ*E*_CR_ energy differences. Excitation energies at the Franck–Condon region are included in the gray rectangle. On the right, the excited-state energies at the estimated minimum-energy geometries to compute Δ*E*_CS_ and Δ*E*_CR_ are shown. Color code: black is used for the ground state, red for CT states (*i.e*., *|*D^+^A^*–*^>), and green for LE states (*|*D^*^A>).

To estimate the free energy difference for the CS and CR processes (Δ*G*_CS_ and Δ*G*_CR_), the optimization of the lowest-energy charge-transfer (CT_1_, CT_2_, and CT_3_) and local (LE_1_, LE_2_, and LE_3_) excited states is required. Figure 4 displays a schematic diagram with the vertical and adiabatic energies calculated for the three lowest-energy CT and LE excited states of the truxTTF·C_30_H_12_ heterodimer in *o*-dichlorobenzene within the SS-PCM approach. Table 2 collects all of the Δ*G*_CS_ and Δ*G*_CR_ values computed for truxTTF·C_30_H_12_, which are further employed for the calculation of the CS and CR rate constants (see below). The Gibbs free energy differences Δ*G* are approximated by assuming to be similar to the adiabatic energy differences Δ*E* (*i.e*., Δ*G*_CS_ ≈ Δ*E*_CS_ and Δ*G*_CR_ ≈ Δ*E*_CR_).^41,44^

**Table 2:** Gibbs Free Energy Differences (Δ*G*) between the Ground State and the CT States and between the LE and CT Excited States Involved in the CR and CS Electronic Processes Computed at the OT-LC-BLYP(ω = 0.16 bohr^–1^)/6–31G** Level in *o*-Dichlorobenzene within the SS-PCM Approximation

Δ*G* (eV)			
	CT_1_	CT_2_	CT_3_
GS	–1.75	–2.02	–2.08
LE_1_	–0.70	–0.43	–0.37
LE_2_	–0.86	–0.59	–0.53
LE_3_	–0.87	–0.60	–0.54

Prior to discussing the adiabatic energy differences Δ*E*_CS_ and Δ*E*_CR_, it is interesting to compare the vertical excitation energies (Frank–Condon region) calculated for the lowest-energy excited states of truxTTF·C_30_H_12_ at the OT-LC-BLYP(ω = 0.16 bohr^–1^)/6-31G** level in *o*-dichlorobenzene within both the linear-response and the state-specific PCM approach. TDA–DFT within the SS-PCM approximation predicts excitation energies of 2.22, 2.29, and 2.35 eV for the GS → CT_1_, GS → CT_2_, and GS → CT_3_ transitions, respectively (Table S11). These transitions are strongly stabilized (by *ca*. 0.4–0.7 eV) when compared to the vertical excitations obtained using the standard linear-response PCM formalism (2.69, 2.88, and 2.95 eV for the GS → CT_1_, GS → CT_2_, and GS → CT_3_ transitions, respectively, Table S8). Note that practically identical excitation energies (2.69, 2.87, and 2.91 eV, respectively) are found when computed in the gas phase. These outcomes highlight the relevance of using the SS-PCM approach, which accounts for the density of the specific state instead of the transition density, to properly capture the expected stabilization of the CT-like excited states by solvent effects (polarization and relaxation). The notorious energy stabilization found for the CT excitations when using the SS-PCM approach is in line with previous studies.^39,86^ The polarization and relaxation solvent effects described by SS-PCM are, therefore, necessary for the correct prediction of Δ*G*_CS_ and Δ*G*_CR_ and, consequently, for the accurate estimation of the CS and CR electron-transfer rate constants. In contrast to the CT-type transitions, the LE excitation energies, for which solvent effects are expected to be less important, barely show differences between the two PCM approaches (Table S11).

We now turn our attention to the adiabatic energies obtained by full-geometry optimization of the low-lying excited states of interest. The CT_1_ state of truxTTF·C_30_H_12_ was initially optimized at the OT-LC-BLYP(ω = 0.16 bohr^–1^)/6-31G** level in the presence of *o*-dichlorobenzene with the linear-response PCM approach. The minimum-energy structure of this CT state displays shorter intermolecular distances compared to the ground-state geometry, in concordance with the enhanced attractive Coulombic interaction between the donor truxTTF and acceptor C_30_H_12_ units in this state. This structural rearrangement in CT_1_ from the ground-state geometry (Franck–Condon region) is accompanied by a relaxation energy of 0.49 eV. The CT_1_ energy at the optimized geometry within the linear-response PCM scheme was additionally refined using the equilibrium SS-PCM approach, to take into account the stabilization due to the environmental effects (∼0.50 eV). The adiabatic energy difference between CT_1_ and GS (Δ*E*_CT1-GS_ = Δ*E*_CR_ ≈ Δ*G*_CR_) was thereby estimated to be 1.75 eV. For the other low-lying CT states (CT_2_ and CT_3_), it was safely assumed that their minimum-energy geometries were similar to that obtained for the CT_1_ state. Consequently, the energy of the CT_2_ and CT_3_ excited states was recalculated with the equilibrium SS-PCM approach at the CT_1_-optimized truxTTF^+^·C_30_H_12_^–^ geometry. The corresponding adiabatic Δ*E*_CT2-GS_ and Δ*E*_CT3-GS_ energies after solvent corrections were estimated to be 2.02 and 2.08 eV, respectively.

The optimization of the local excited states (LE_1_, LE_2_, and LE_3_) of the supramolecular truxTTF·C_30_H_12_ heterodimer was less feasible. Convergence problems in excited-state optimizations often appear when there is a manifold of excited states of similar nature in a narrow energy window. To circumvent this technical issue, and considering that these excitations are totally centered on the donor truxTTF unit, the energies of the LE_1_, LE_2_, and LE_3_ minima were estimated by correcting the vertical excitation energies Δ*E*_exc_ at the ground-state geometry of truxTTF·C_30_H_12_ with the relaxation energies Δ*E*_rel_ obtained from the optimization of the three first singlet excited states of the isolated truxTTF moiety (0.30, 0.46, and 0.47 eV for LE_1_, LE_2_, and LE_3_, respectively). The estimated adiabatic energy differences for the local excited states Δ*E*_LE1–GS_, Δ*E*_LE2–GS_, and Δ*E*_LE3–GS_ were then calculated to be 2.45, 2.61, and 2.62 eV, respectively (Figure 4).

In line with the picture found at the Franck–Condon region, Figure 4 clearly shows that there are two sets of excited states well-separated in energy: the three lowest-energy CT excited states and the LE excitations, LE_1_ being an almost dark state and LE_2_ and LE_3_ being bright states (Table S11). As the energy difference between LE_2_/LE_3_ and LE_1_ local excited states is small (<0.2 eV), an internal conversion from the bright states to the LE_1_ state is likely to take place. The CS process can thus occur from this LE_1_ state to any CT state (CT_1–3_). Finally, the CR process is meant to occur from the lowest-energy CT_1_ state, after internal conversion, to the ground state. Nonetheless, the CS and CR rate constants for all of the possible charge-transfer pathways were computed as detailed below.

### Internal and External Reorganization Energy

The internal reorganization energy λ_int_ of the CS and CR processes (λ_int_^CS^ and λ_int_^CR^) was computed according to eqs S5 and S6, respectively, for which the energies calculated for the isolated truxTTF and C_30_H_12_ compounds are employed (Figure S5). The energies of the donor and acceptor species were computed using the OT-LC-BLYP density functional with optimized ω values of 0.03 and 0.04 bohr^–1^ for truxTTF and C_30_H_12_, respectively. For the CS process, the internal reorganization components of the truxTTF and C_30_H_12_ units were calculated to be 0.50 and 0.06 eV, respectively, being λ_int_^CS^ = 0.56 eV. For CR, a smaller λ_int_^CR^ value of 0.13 eV is predicted, with internal reorganization components of 0.07 and 0.06 eV for truxTTF and C_30_H_12_, respectively. A quick comparison of the λ_int_^CS^ and λ_int_^CR^ values reveals that the energy difference between the two magnitudes (0.43 eV) mainly comes from the donor truxTTF unit and is due to the difference between the equilibrium structures obtained for truxTTF in its excited state and in its cation/neutral ground state (Figure S6). In contrast, the internal reorganization energy components for C_30_H_12_ in both CS and CR processes present a similar and small value (0.06 eV) due to the rigidity of the C_30_H_12_ buckybowl.

**Figure 5:**
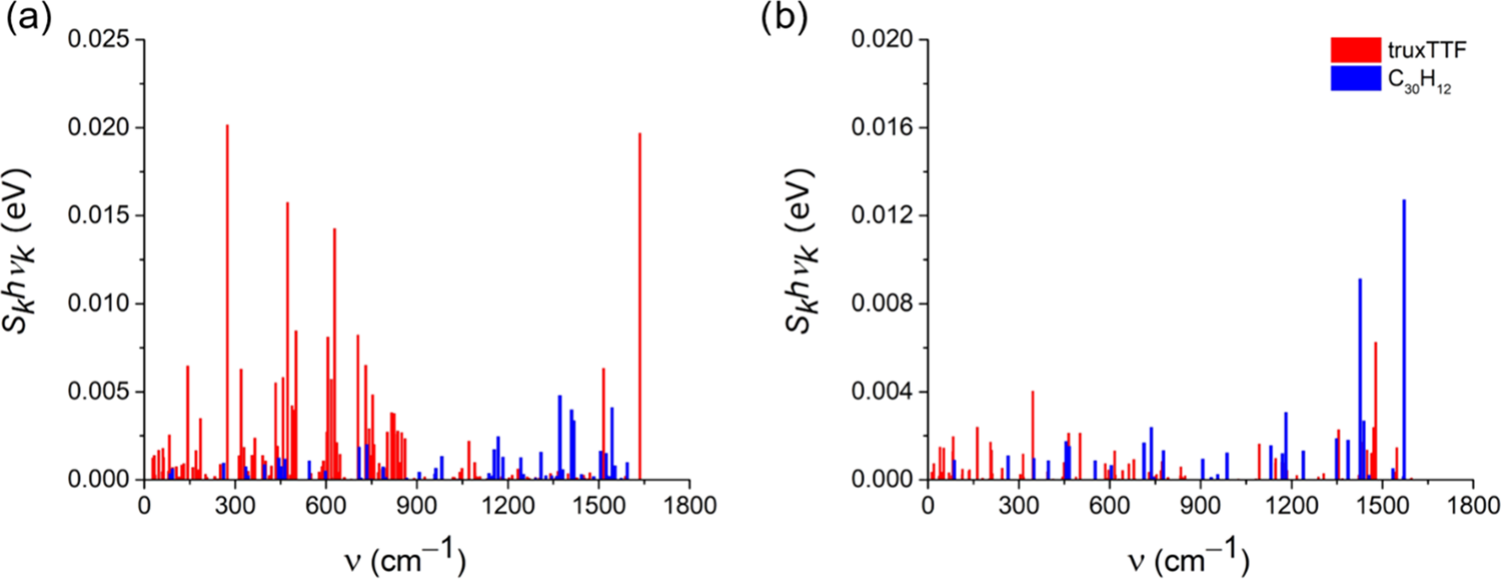
Contribution of each normal mode to the internal reorganization energy of (a) the charge-separation and (b) charge-recombination processes calculated at the OT-LC-BLYP/6-31G** level, with ω values of 0.03 and 0.04 bohr^–1^ for the isolated truxTTF and C_30_H_12_ compounds, respectively.

The internal reorganization energies of the CS and CR events have been additionally decomposed in contributions for each vibrational normal mode by calculating the HR factors according to Malagoli et al.^87^ (see the Supporting Information for further details). Figure 5 displays the decomposition of λ_int_^CS^ and λ_int_^CR^ in the vibrational modes of the isolated truxTTF and C_30_H_12_ compounds calculated at their respective OT-LC-BLYP/6–31G** level. For CS (Figure 5a), truxTTF possesses many active vibrations along the frequency spectrum. Among them, there are four normal modes calculated at 274, 473, 628, and 1636 cm^–1^ showing especially large contributions to λ_int_^CS^. The low-frequency vibrations (below 1000 cm^–1^) correspond to either the bendings of the truxTTF core or rotations of the dithiole rings (Figure S6a), whereas the high-frequency mode (1636 cm^–1^) is related to the stretching of single and double carbon–carbon (C–C/C=C) bonds of the π-conjugated truxTTF skeleton. For the C_30_H_12_ hemifullerene, four high-frequency vibrations computed at 1371, 1410, 1418, and 1544 cm^–1^ and associated with C–C/C=C bond stretchings are responsible for the highest contributions to λ_int_^CS^. For CR (Figure 5b), the λ_int_^CR^ decomposition is much simpler compared to the CS process, presenting only three high-frequency vibrations with significant contributions: one active normal mode for truxTTF (1478 cm^–1^) and two vibrations for C_30_H_12_ (1426 and 1571 cm^–1^). These normal modes are also described by stretchings of C–C/C=C bonds.

All frequencies higher than 250 cm^–1^ were treated quantum mechanically and condensed in an effective vibration. The frequency for this effective vibration was computed as *ν*_eff_ = ∑_ν_*k*_>250_*S*_*k*_ν_*k*_/∑_ν_*k*_>250_*S*_*k*_, giving a *ν*_eff_ value of 683 cm^–1^ for the CS process. A quantum internal reorganization energy contribution for CS (λ_int,q_^CS^) can be defined as λ_int,q_^CS^ = ∑_ν_*k*_>250_*hν*_*k*_*S*_*k*_ with a value of 1816 cm^–1^. This λ_int,q_^CS^ contribution must be recovered by the reorganization energy inferred from the effective vibration (*i.e*., λ_int,q_^CS^ = *hν*_eff_*S*_eff_). From the latter expression, the effective HR factor *S*_eff_ can then be evaluated (2.66). The computed λ_int,q_^CS^ value of 0.23 eV represents 41% of the total internal reorganization energy for the CS process discussed above (λ_int_^CS^ = 0.56 eV). The remaining part of the internal reorganization energy, computed as the difference between λ_int_^CS^ and λ_int,q_^CS^ (λ_int,c_^CS^ = λ_int_^CS^ – λ_int,q_^CS^ = 0.34 eV), is treated classically and included together with the external reorganization energy in λ_c_ (λ_c_ = λ_int,c_^CS^ + λ_ext_^CS^) in the final rate expression (eq 1). A similar procedure was adopted for the CR process. An effective frequency of 872 cm^–1^ and a *S*_eff_ = 0.97 were estimated with a quantum internal reorganization energy λ_int,q_^CR^ of 0.10 eV, which is 77% of the internal reorganization energy for that electron-transfer process (0.13 eV). The classical internal reorganization energy λ_int,c_^CR^ is therefore computed to be very small (0.03 eV).

Regarding the external reorganization energy λ_ext_, different values are expected for the CS and CR processes (λ_ext_^CS^ and λ_ext_^CR^) since the solvent molecules should reorient their positions in response to the different electronic situations. For CS, the solvent molecules should undergo significant polarization and reorganization due to the charged truxTTF^+^·C_30_H_12_^–^ complex, whereas the rearrangement of the solvent molecules surrounding the neutral truxTTF·C_30_H_12_ after CR should be smaller. As mentioned above, λ_ext_ was computed using the “nonequilibrium vs equilibrium solvation” model within the SS-PCM approach (see the Supporting Information for additional details).^39^ Briefly, the λ_ext_^CS^ and λ_ext_^CR^ components were estimated as the energy difference between the total reorganization energy λ, computed according to eqs S9–S12 for the isolated fragments, and the corresponding internal λ_int_^CS^ and λ_int_^CR^ contributions. For CS, the λ_ext_^CS^ is estimated to be 0.89 eV, with 0.38 and 0.51 eV for the truxTTF and C_30_H_12_ fragment contributions, respectively. A slightly smaller λ_ext_^CR^ value of 0.72 eV is found for the CR event, with contributions of 0.28 and 0.44 eV for the truxTTF and C_30_H_12_ moieties, respectively. Note that the λ_ext_^CS^ and λ_ext_^CR^ values are significantly larger than those calculated for the internal reorganization energy, and thus, λ_ext_ has a larger impact on the calculation of CS and CR rate constants, in contrast to what occurs in molecular crystals where λ_ext_ is generally small.^88^

As λ_ext_ is estimated from an energy difference between λ and λ_int_^CS^ or λ_int_^CR^ (eq S8) at different geometries, the dependence of λ_ext_ with respect to the molecular structure has also been analyzed. To do so, λ_ext_ at a fixed geometry (ground-state geometry for the neutral fragments) is calculated for both charge-transfer CS and CR events. For CS, the nonequilibrium energy component was calculated using the density obtained from the LE_1_ excited state/ground state for truxTTF*/C_30_H_12_, whereas for the equilibrium energy contribution, the density calculated for the cationic/anionic states of truxTTF^+^/C_30_H_12_^–^ was used. An external reorganization energy of 0.37 eV (0.44 eV) for truxTTF (C_30_H_12_) was obtained, providing a total external reorganization energy of 0.81 eV. For CR, the external reorganization energy was now computed using the cation/anion densities as the nonequilibrium components and the ground-state density as the equilibrium component. The resulting λ_ext_ values were found to be 0.32 and 0.44 eV for truxTTF and C_30_H_12_, respectively, being the total external reorganization energy of 0.76 eV. The similarity between the external reorganization energy values computed at a fixed geometry (0.81 and 0.76 eV for CS and CR, respectively) and the λ_ext_^CS^ and λ_ext_^CR^ values calculated above (0.89 and 0.72 eV) indicates that there is a small influence of the internal molecular structure on the solvent reorganization energy.

### Charge-Separation (*k*_CS_) and Charge-Recombination (*k*_CR_) Rates

**Figure 6:**
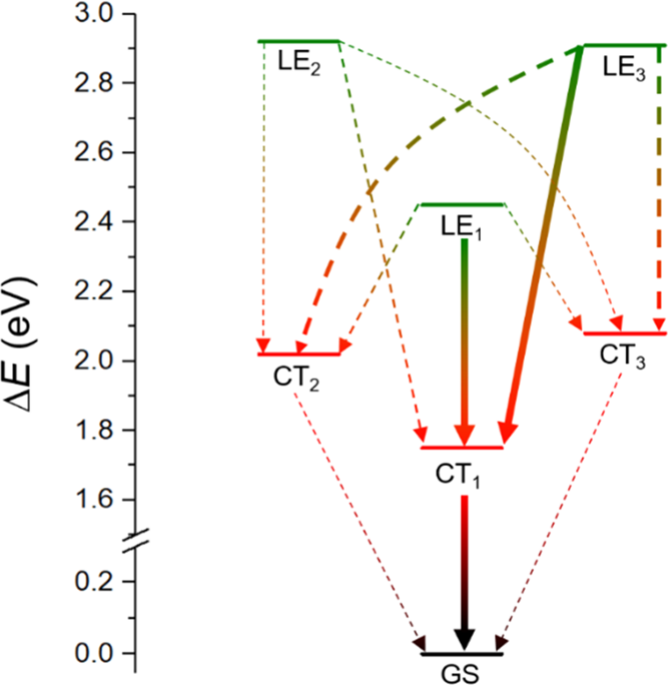
Scheme for all of the CS and CR pathways. The thickness of the arrows indicates the relevance of the decay channels according to Table 3.

In the previous sections, all of the parameters needed to estimate the electron-transfer rates using the MLJ equation (eq 1) have been computed and discussed. Table 3 presents all of the relevant parameters and the values computed for the *k*_CS_ and *k*_CR_ rate constants of the different electron-transfer pathways in the truxTTF·C_30_H_12_ heterodimer, whereas Figure 6 shows a schematic picture of the kinetical relevance of the different electron-transfer channels. For CS, the fastest electron-transfer rate constants are found to be those occurring from the initial LE_1_ (S_4_) and LE_3_ (S_6_) excited states to the final CT_1_ (S_1_) state with CS rates of 2.0 × 10^12^ and 3.0 × 10^12^ s^–1^, respectively, which are in good agreement with the reported experimental rate of 6.6 × 10^11^ s^–1^.^23^ Although our results indicate that the most probable pathway for charge separation is from LE_3_ to CT_1_, an ultrafast internal conversion from LE_3_ to LE_1_ together with a slower LE_1_ → CT_1_ CS transition would also be feasible. After the CS event, CR takes place from the lowest-energy CT_1_ state to the ground state with a CR rate constant *k*_CR_ of 2.6 × 10^9^ s^–1^, also in reasonably good accord with the experimentally estimated rate constant (1.0 × 10^10^ s^–1^).^23^ The constant rates for the most relevant pathways (LE_1_ → CT_1_, LE_3_→ CT_1_, and CT_1_→ GS) have also been computed with the Marcus theory for comparison purposes (see the Supporting Information for further details).

**Table 3:** Relevant Parameters (*V*_*ij*_, Δ*G*_*ij*_, λ_c_, *S*_eff_, and *ν*_eff_, in eV) and Estimated *k*_CS_ and *k*_CR_ Rate Constants (in s^–1^) for the Different Electron-Transfer Pathways in the Donor-Acceptor truxTTF·C_30_H_12_ Heterodimer

transition	*V*_*ij*_	–Δ*G*_*ij*_	λ_c_	*S*_eff_	*ν*_eff_^a^	*k*_*ij*_
CS process						
LE_1_ → CT_1_	0.044	0.74	1.23	2.66	0.085 (683)	2.0 × 10^12^
LE_1_ → CT_2_	0.020	0.47	1.23	2.66	0.085 (683)	2.2 × 10^10^
LE_1_ → CT_3_	0.015	0.41	1.23	2.66	0.085 (683)	4.7 × 10^9^
LE_2_ → CT_1_	0.004	0.83	1.23	2.66	0.085 (683)	3.8 × 10^10^
LE_2_ → CT_2_	0.006	0.56	1.23	2.66	0.085 (683)	6.0 × 10^9^
LE_2_ → CT_3_	0.009	0.50	1.23	2.66	0.085 (683)	6.6 × 10^9^
LE_3_ → CT_1_	0.033	0.84	1.23	2.66	0.085 (683)	3.0 × 10^12^
LE_3_ → CT_2_	0.024	0.57	1.23	2.66	0.085 (683)	1.3 × 10^11^
LE_3_ → CT_3_	0.009	0.51	1.23	2.66	0.085 (683)	1.0 × 10^10^
CR process						
CT_1_ → GS	0.010	1.75	0.75	0.97	0.108 (872)	2.6 × 10^9^
CT_2_ → GS	0.001	2.02	0.75	0.97	0.108 (872)	1.7 × 10^5^
CT_3_ → GS	0.004	2.08	0.75	0.97	0.108 (872)	2.3 × 10^4^

a Values within parentheses are in cm^–1^.

Finally, to analyze the effect of the supramolecular organization on the CS and CR rates, the electron-transfer processes were evaluated for the minimum-energy structures **2** and **3** of the truxTTF·C_30_H_12_ heterodimer displayed in Figure 2. The values computed for the relevant parameters and the rate constants of **2** and **3**, respectively, are presented in Tables S12 and S13. Structure **1** in Figure 2 does not present favorable charge-transfer processes as discussed below. The highest *k*_CS_/*k*_CR_ rates for structures **2** and **3** are calculated to be 1.4 × 10^11^/4.3 × 10^9^ and 8.6 × 10^10^/1.7 × 10^8^ s^–1^, respectively. The fastest CS events are, therefore, found for structure **4** (2.0 and 3.0 × 10^12^ s^–1^), which exhibits electronic states with high electronic couplings and Δ*G*_CS_ values near the resonance with respect to the reorganization energy (see Table 3 and Tables S12 and S13). A closer analysis of the adiabatic excitation energies for all of the structures (Table S14) highlights that those supramolecular arrangements with C···S intermolecular interactions, irrespective of the staggered or bowl-in-bowl organization (*i.e*., structures **2** and **4**), tend to stabilize a larger number of CT-type excited states below the lowest-energy LE excited states, thus opening the door for different and efficient charge-separation pathways. Structure **3**, which is a staggered arrangement with no C···S interactions, presents only one accessible CT excited state below the LE states for a favorable CS event. Surprisingly, structure **1** with a bowl-in-bowl disposition and optimal π-π interactions made only by C···C intermolecular contacts does not present CT excited states below the lowest-energy LE states and, consequently, no photoinduced electron-transfer is expected for this supramolecular structure. Finally, the CR process takes place in the inverted Marcus regime with similar *k*_CR_ rate constants around (3–4) × 10^9^ s^–1^ for structures **2** and **4** and one order of magnitude slower for structure **3**.

### Charge-Separation and Charge-Recombination Kinetic Model

**Figure 7:**
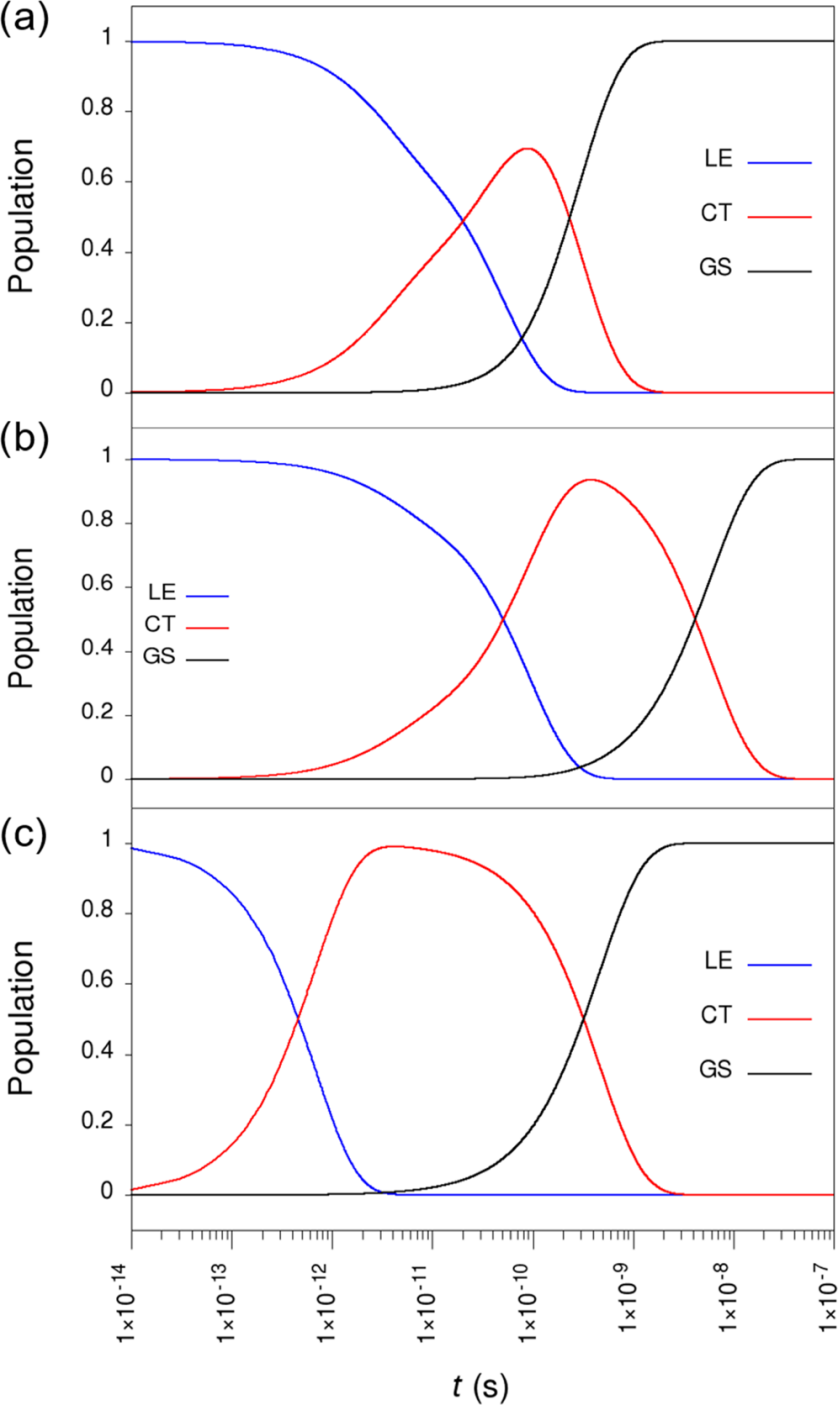
Time evolution of the populations of the electronic states according to their nature (LE, CT, and GS) calculated for structures **2** (a), **3** (b), and **4** (c) according to the kinetic model proposed (see the Supporting Information for further details). Time (*x-*axis) is represented in the logarithmic scale.

To obtain a global picture of the CS and CR electronic events for structures **2**, **3**, and **4**, a simple kinetic model including all of the previously computed rate constants for the different decay pathways was built (see the Supporting Information for further details). Figure 7 displays a global representation of the time evolution of the populations of the electronic states according to their nature (LE, CT, and GS) calculated for structures **2**, **3**, and **4**, whereas Figure S8 shows the populations for each particular excited state as a function of time. A detailed inspection of Figure 7 reveals that the fastest CS electron-transfer process occurs for structure **4**. Actually, a decrease of 50% (99%) in population for LE states is achieved after 18.8 (215), 50.0 (416), and 0.5 (2.9) ps for structures **2**, **3**, and **4**, respectively. Nevertheless, it should be noted that the nonradiative CS mechanism for structures **2**, **3**, and **4** is different (Figure S8). For structures **2** and **3**, the CS deactivation pathway occurs from the LE_1_ state after a faster downhill internal conversion from the bright states (LE_2_ and LE_3_ states). On the contrary, a direct charge separation takes place from the bright states (LE_2_ and LE_3_) for structure **4** because the LE_3_ → CT_1_ charge separation rate is competitive with the nonradiative LE_3_ → LE_1_ internal conversion. For CR, an increase in the population of 50% for the ground state is calculated to occur at 0.24, 4.16, and 0.32 ns for structures **2**, **3**, and **4**, respectively. Interestingly, the half-life times estimated from populations of the LE, CT, and GS states are used to compute global effective rates for the CS and CR processes in a more intuitive and simplified three-state picture. Our kinetic model leads to global CS (CR) rate constants of 5.3 × 10^10^, 2.0 × 10^10^, and 2.0 × 10^12^ s^–1^ (4.3 × 10^9^, 2.4 × 10^8^, 3.1 × 10^9^ s^–1^) for structures **2**, **3**, and **4**, respectively. Note that the theoretical global rates calculated for structure **4** (the most stable) are in good accord with the global experimental values estimated from spectroscopic measurements (6.6 × 10^11^ and 1.0 × 10^10^ s^–1^ for CS and CR events, respectively).

## Conclusions

In this work, we have proposed a theoretical protocol to accurately predict charge-separation (CS) and charge-recombination (CR) rate constants for a donor–acceptor (D–A) buckybowl-based supramolecular complex (truxTTF·C_30_H_12_). The computational approach combines the Marcus–Levich–Jortner (MLJ) rate expression together with electronic structure calculations (at the DFT and TDDFT level) and a multistate diabatization method (an extended fragment charge difference scheme^58^) to carefully calculate the different terms entering into the rate expression (*i.e*., electronic couplings, reorganization energy, and Gibbs free energy difference). Our results clearly disclose that optimally tuned (OT) long-range corrected (LC) density functionals are necessary to provide a correct energy ordering of the low-lying excited states. The OT-LC-BLYP predicts a complex scenario with at least six, low-lying, close-in-energy excited states of local and CT character potentially involved in the CS and CR processes. In this context, which can be generally found in many other D–A heterojunctions, the inclusion of multistate effects is shown to have a strong impact on the accurate estimation of the electronic couplings. We also demonstrate the relevance of the correct stabilization of the CT states due to the solvent effect, accounted using the state-specific PCM solvation model. After the careful estimation of all of the specific CS and CR rate constants for the different deactivation pathways, a simple but insightful kinetic model is proposed to estimate the global CS and CR rate constants in an effective three-state picture. The values computed for the global CS and CR rates of the donor–acceptor truxTTF·C_30_H_12_ supramolecular complex are found to be in good agreement with the experimental values. The suggested theoretical protocol including multistate effects and an accurate state-specific description of the solvent effects is of general application to any other D–A molecular or supramolecular system with potential for organic solar cells.

## Supporting Information Available

Additional theoretical details, excited-state analysis, estimation of reorganization energies, excitations and electronic couplings for structures **1**, **2**, and **3**, and details about the kinetic model (PDF)
