# Supplementary material for: Charge-Separation and Charge-Recombination Rate Constants in a Donor–Acceptor Buckybowl-Based Supramolecular Complex: Multistate and Solvent Effects
Source: J Phys Chem A. 2021 Nov 12;125(46):9982–94. doi: 10.1021/acs.jpca.1c05740 (PMC8630798; doi:10.1021/acs.jpca.1c05740)
Supplement: Supplementary file 1 — jp1c05740_si_001.pdf [file jp1c05740_si_001.pdf]

Supporting Information

Charge-Separation and Charge-Recombination Rate Constants in a Donor-Acceptor Buckybowl-based Supramolecular Complex: Multi-State and Solvent Effects

*Jesús Cerdá[a], Joaquín Calbo[a], Enrique Ortí[a], and Juan Aragó[a]**

[a]*Instituto de ciencia molecular (ICMol), Universidad de Valencia, Catedrático José Beltrán 2, Paterna, 46980, Spain*

**Email: juan.arago@uv.es*

Contents

S1. Theoretical Details **3**

S1.1 Franck−Condon Integral 3

S1.2 The Two-State and the Multi-State FCD Methods 3

S1.3 Tunning of the Long-Range Corrected Density Functionals 5

S2. Excited State Analysis **8**

S2.1 DFT Comparison 8

S2.2 Low-Lying Excited States at OT-LC-BLYP(ω = 0.16 Bohr^{-1}) 14

S3. Internal Reorganization Energy and Huang-Rhys Factors **17**

S4. External Reorganization Energy **21**

S5. Analysis of truxTTF•$C_{30}H_{12}$ Supramolecular Structures **22**

S6. Kinetic Model **24**

S7. References **26**

S1. Theoretical Details

S1.1 Franck−Condon Integral

$$FCI_{nm}(S_{\mathrm{eff}}) = \exp\left(-\frac{S_{\mathrm{eff}}}{2}\right)\sum_{u=0}^{n}\sum_{w=0}^{m}\frac{(-1)^{w}\left(\sqrt{S_{\mathrm{eff}}}\right)^{u+w}}{u!w!}\sqrt{\frac{n!m!}{(n-u)!(m-w)!}}\delta_{n-u,m-w} \qquad \text{Eq. S1}$$

As stated in the main text, $FCI_{nm}\left(S_{\mathrm{eff}}\right)$ denotes the Franck−Condon integral between the initial (*n*) and final (*m*) vibrational levels of the initial *i* and final *j* electronic states.

S1.2 The Two-State and the Multi-State FCD Methods

In the two-state fragment charge difference (FCD) scheme, the whole molecular system (here the truxTTF•$C_{30}H_{12}$ heterodimer) is divided into two fragments (the electron-donor truxTTF and the electron-acceptor $C_{30}H_{12}$) and a 2 × 2 donor–acceptor charge difference matrix ($\Delta\mathbf{q}$) is defined as

$$\Delta\mathbf{q} = \begin{bmatrix} \Delta q_{ii} & \Delta q_{ij} \\ \Delta q_{ij} & \Delta q_{jj} \end{bmatrix} \qquad \text{Eq. S2}$$

The Δq_{ij} matrix elements are calculated from

$$\Delta q_{ij} = \int_{\mathbf{r}\in \mathrm{D}} \rho_{ij}(\mathbf{r})\mathrm{d}\mathbf{r} - \int_{\mathbf{r}\in \mathrm{A}} \rho_{ij}(\mathbf{r})\mathrm{d}\mathbf{r} \qquad \text{Eq. S3}$$

where ρ_{ij} is the one-particle density ($i = j$), or the transition density between the initial i and final j electronic states for the donor–acceptor (D−A) system.

Table S1 shows a schematic form of the Hamiltonian and FCD matrices in the adiabatic/diabatic representation. Similar to the Generalized Mulliken−Hush method,[1] the FCD scheme assumes a diagonal charge difference matrix ($\Delta\mathbf{q}$) in the diabatic representation and, therefore, the off-diagonal elements are zero (Table S1). In the case of the charge recombination process in a D−A supramolecular complex (D^{+}−A^{-} → D−A), the ideal (diabatic) initial state would present a Δq_{11} value of 2 since the D fragment (D^{+}) has a net charge of +1e whereas the A fragment (A^{-}) holds a net charge of −1e. The ideal final state (D−A ground state) would have a Δq_{22} value of zero since both the D and A fragments are neutral. The diabatic off-diagonal Δq_{12} and Δq_{12} elements are zero because there is no overlap between the diabatic initial and final states. Once the adiabatic $\Delta\mathbf{q}$ matrix is evaluated at any quantum chemistry level (the corresponding diabatic $\Delta\mathbf{q}$ matrix is already known), the adiabatic-to-diabatic unitary transformation $\mathbf{C}$ can be calculated. The diabatic Hamiltonian can be then computed by applying a unitary transformation to the known adiabatic Hamiltonian ($\mathbf{H}^{\mathbf{DI}} = \mathbf{C}\mathbf{H}^{\mathbf{AD}}\mathbf{C}^{T}$). In the diabatic

$\mathbf{H}^{\mathrm{DI}}$ Hamiltonian matrix, the diagonal elements correspond to the diabatic energies (ε_i and ε_j) whereas the off-diagonal elements are the desired electronic couplings (V_{ij}).

Table S1. Schematic representation of the matrices involved in the FCD diabatization method.

Matrix	Adiabatic	Diabatic
Hamiltonian, **H**	$\begin{pmatrix} E_i & 0 \\ 0 & E_j \end{pmatrix}$	$\begin{pmatrix} \varepsilon_i & V_{ij} \\ V_{ji} & \varepsilon_j \end{pmatrix}$
Charge difference, $\Delta\mathbf{q}$	$\begin{pmatrix} \Delta q_{ii} & \Delta q_{ij} \\ \Delta q_{ji} & \Delta q_{jj} \end{pmatrix}$	$\begin{pmatrix} \Delta q_i & 0 \\ 0 & \Delta q_j \end{pmatrix}$

The multi-state variant of the FCD diabatization scheme is based on the same principle as the two-state model, but using an $N \times N$ matrix where N is the number of diabatic/adiabatic states. The algorithm is similar to that used in the two-state model but it requires the diagonalization of the blocks corresponding to local (LE) and charge-transfer (CT) excitations to ensure zero couplings between the electronic states of similar nature (see the work of Yang and Hsu for further details).[2]

S1.3 Tunning of the Long-Range Corrected Density Functionals

Long-range corrected (LC) functionals split the exchange term in a short-range and a long-range component. The first component is represented by the exchange expression of

the own functional whereas the second is evaluated with an “exact” Hartree-Fock exchange expression.[3] LC functionals significantly depend on the ω parameter, which measures the threshold distance for the short-range and long-range region. Optimization of the ω parameter for an LC functional applied to a donor−acceptor (D−A) supramolecular complex is highly recommended to obtain acceptable energies for the CT excited states and satisfy the Mulliken rule $E_{CT} = IP + EA - 1/r$,[3] where IP, EA, and r correspond to the ionization potential, the electron affinity, and the distance between the donor and acceptor units, respectively. Here, we have performed an optimization of ω by minimizing the $J(\omega)$ function (Eq. S4) that provides the deviation of the HOMO energy ($\varepsilon_{HOMO}^{\omega,S}$) of the neutral system with respect to the IP plus the deviation of the HOMO energy of the anion system with respect to the EA.

$$J(\omega) = \left|\varepsilon_{HOMO}^{\omega,S} + IP\right| + \left|\varepsilon_{HOMO}^{\omega,S^-} + EA\right| \qquad \text{Eq. S4}$$

Figure S1 displays the plot of $J(\omega)$ as a function of ω for the truxTTF and hemifullerene $C_{30}H_{12}$ fragments using the LC-BLYP functional. Optimal ω parameters of 0.03 and 0.04 Bohr^{-1} with $J(\omega)$ values of 0.070 and 0.049 eV were obtained for the truxTTF and $C_{30}H_{12}$ moieties, respectively. These small ω values mean that in monomers the long-range correction is only applied at long distances, being the main exchange contribution from

the pure functional. For instance, the optimum ω value for truxTTF means than the long-range term is employed for electron-electron interactions with a distance higher than 17.64 Å. Taking into account that the length of the molecule is around 14.00 Å, the long-range term in the LC approximation has a small impact on the isolated monomers.

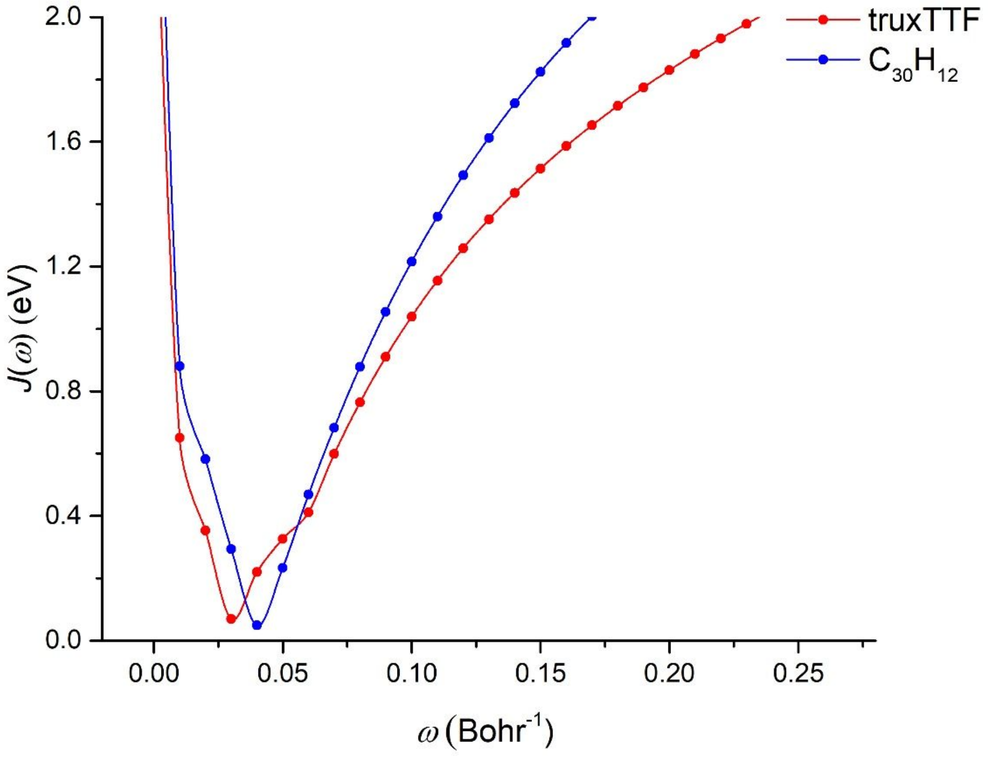

Figure S1. Plot of the $J(\omega)$ function for truxTTF and $C_{30}H_{12}$ using the LC-BLYP functional and the 6-31G** basis set.

Optimization of ω was also performed for all the minimum-energy structures (structures **1**−**4** in Figure 1) of the truxTTF•$C_{30}H_{12}$ heterodimer (Figure S2). The optimized ω values

for structures **1–4** were found to be 0.18, 0.17, 0.14, and 0.16 Bohr^{-1} with $J(\omega)$ values of 0.34, 0.13, 0.012, and 0.099 eV, respectively. Higher ω values were therefore obtained for the supramolecular truxTTF•$C_{30}H_{12}$ complex than for the isolated monomers. This emphasizes that the LC approximation is more relevant for the supramolecular complex.

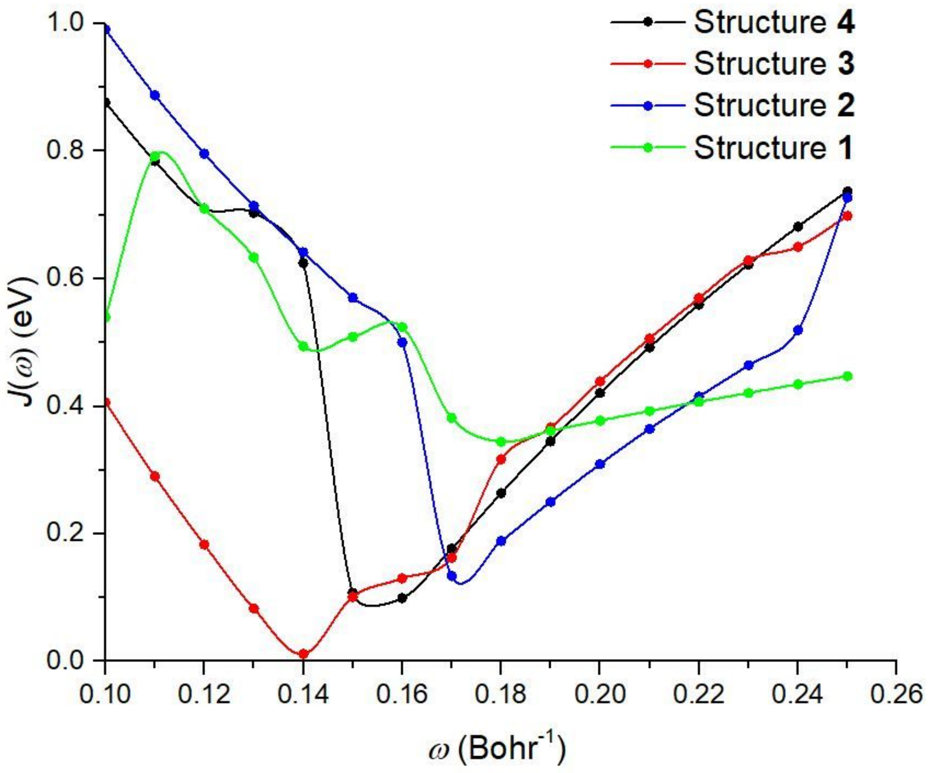

Figure S2. Plot of the $J(\omega)$ function for the minimum-energy structures **1−4** of the truxTTF•$C_{30}H_{12}$ heterodimer using the LC-BLYP functional and the 6-31G** basis set.

S2. Excited State Analysis

S2.1 DFT Comparison

Although in our previous study[4] B3LYP provided a satisfactory description of the absorption spectrum of the truxTTF•$C_{30}H_{12}$ supramolecular heterodimer, a large number of CT states appeared significantly stabilized due to the well-known self-interaction errors.[5] Here, a correct description (excitation energies) of CT excited states is crucial to accurately estimate the CS and CR rate constants, and the performance of several LC functionals (LC-BLYP, CAM-B3LYP, LC-ωPBE, and ωB97X-D) together with their optimally-tuned analogues (OT-LC-BLYP, OT-LC-ωPBE, and OT-ωB97X-D) has been explored. Tables S2-S10 collect the vertical excitations energies and the electronic nature calculated for the lowest-energy singlet excited states using the different DFT functionals. Excitations with Δq values above 1e indicate a significant CT character, whereas excitations with Δq values smaller than 0.5e are characteristic of local excitations involving only the truxTTF donor. Δq values between 0.5 and 1e correspond to states with a mixed character (*i.e.*, LE&CT).

As expected, BLYP and B3LYP functionals provide a complicated picture of the lowest-energy excited states, with many excited states of CT nature below the local bright

excited states (Table S2 and S3). These findings are in line with the trend of GGA and hybrid density functionals that usually underestimate the energy positions of CT excitations owing to the well-known self-interaction errors.[5–8] In contrast, the standard LC density functionals LC-BLYP, LC-ωPBE, ωB97X-D, and CAM-B3LYP (Tables S4–S7) predict the lowest-energy CT excited states above in energy compared to the LE states. This excited-state ordering would not promote a favorable photoinduced electron transfer, and does not agree with the experimental absorption spectra of truxTTF•$C_{30}H_{12}$, where a CT absorption band is observed lower in energy than the intense absorption band associated to the donor truxTTF (2.76 eV, 450 nm).[4] Optimally tuned long-range corrected functionals yield a more reasonable description of the excited states (Tables S8−S10). Among them, the OT-LC-BLYP(ω = 0.16 Bohr^{-1}) functional shows the best performance; the bright LE states present excitation energies (3.04−3.06 eV, 405−408 nm) in reasonably good agreement with the truxTTF absorption spectrum (2.76 eV) together with a few CT states lower in energy than the bright LE states, thus enabling a favorable photoinduced electron transfer pathway (Table S8). Additionally, all the low-lying excited states can be well characterized as LE- or CT-type states, and thus considered as quasi-diabatic states, which is appropriate for the satisfactory employment of non-adiabatic charge-transfer rate constants. Therefore, OT-LC-BLYP(ω = 0.16 Bohr^{-}

[1]) was adopted as the density functional used for the calculation of the parameters related to the estimation of the CS and CR rate constants.

Table S2. Vertical excitation energies (ΔE, in eV) and oscillator strengths (f) for the lowest-energy singlet excited states of the supramolecular D−A truxTTF•$C_{30}H_{12}$ complex calculated at the B3LYP/6-31G** level in *o*-dichlorobenzene (PCM). The Δq values (elementary charge in e) of the corresponding excited states and their electronic nature (LE, CT, or LE&CT) are also provided.

State	ΔE	f	Δq	Nature
S_1	2.366	0.008	1.838	CT
S_2	2.452	0.036	1.706	CT
S_3	2.478	0.004	1.905	CT
S_4	2.554	0.015	0.451	LE
S_5	2.598	0.005	1.723	CT
S_6	2.686	0.015	1.570	CT
S_7	2.712	0.004	1.949	CT
S_8	2.823	0.029	1.121	CT
S_9	2.840	0.188	0.516	LE&CT
S_{10}	2.840	0.046	0.470	LE

Table S3. Vertical excitation energies (ΔE, in eV) and oscillator strengths (f) for the lowest-energy singlet excited states of the supramolecular D−A truxTTF•$C_{30}H_{12}$ complex calculated at the BLYP/6-31G** level in *o*-dichlorobenzene (PCM). The Δq values (elementary charge in e) of the corresponding excited states and their electronic nature (LE, CT, or LE&CT) are also provided.

State	ΔE	f	Δq	Nature
S_1	1.699	0.003	1.939	CT
S_2	1.783	0.000	1.970	CT
S_3	1.816	0.008	1.943	CT
S_4	1.847	0.034	1.883	CT
S_5	1.898	0.000	1.958	CT
S_6	1.923	0.004	1.910	CT
S_7	1.985	0.002	1.955	CT
S_8	2.101	0.004	1.940	CT
S_9	2.111	0.012	0.051	LE
S_{10}	2.123	0.006	1.854	CT
S_{11}	2.307	0.033	0.013	LE
S_{12}	2.356	0.171	−0.039	LE

Table S4. Vertical excitation energies (ΔE, in eV) and oscillator strengths (f) for the lowest-energy singlet excited states of the supramolecular D−A truxTTF•$C_{30}H_{12}$ complex calculated at the LC-BLYP/6-31G** level in *o*-dichlorobenzene (PCM). The Δq values (elementary charge in e) of the corresponding excited states and their electronic nature (LE, CT, or LE&CT) are also provided.

State	ΔE	f	Δq	Nature
S_1	3.616	0.083	0.007	LE
S_2	3.704	0.621	0.001	LE

S_3	3.736	0.521	−0.001	LE
S_4	3.783	0.306	0.000	LE
S_5	3.967	0.003	0.049	LE
S_6	4.160	0.018	0.171	LE

Table S5. Vertical excitation energies (ΔE, in eV) and oscillator strengths (f) for the lowest-energy singlet excited states of the supramolecular D−A truxTTF•$C_{30}H_{12}$ complex calculated at the CAM-B3LYP/6-31G** level in *o*-dichlorobenzene (PCM). The Δq values (elementary charge in e) of the corresponding excited states and their electronic nature (LE, CT, or LE&CT) are also provided.

State	ΔE	f	Δq	Nature
S_1	3.154	0.128	0.141	LE
S_2	3.246	0.632	0.378	LE
S_3	3.368	0.598	0.028	LE
S_4	3.377	0.493	0.042	LE
S_5	3.419	0.101	1.374	CT
S_6	3.462	0.027	1.083	CT

Table S6. Vertical excitation energies (ΔE, in eV) and oscillator strengths (f) for the lowest-energy singlet excited states of the supramolecular D−A truxTTF•$C_{30}H_{12}$ complex calculated at the LC-ωPBE/6-31G** level in *o*-dichlorobenzene (PCM). The Δq values (elementary charge in e) of the corresponding excited states and their electronic nature (LE, CT, or LE&CT) are also provided.

State	ΔE	f	Δq	Nature
S_1	3.532	0.863	0.015	LE
S_2	3.609	0.478	–0.005	LE
S_3	3.650	0.478	–0.006	LE
S_4	3.684	0.434	–0.002	LE
S_5	3.878	0.003	0.066	LE
S_6	4.091	0.039	0.453	LE

Table S7. Vertical excitation energies (ΔE, in eV) and oscillator strengths (f) for the lowest-energy singlet excited states of the supramolecular D−A truxTTF•$C_{30}H_{12}$ complex calculated at the *ω*B97X-D/6-31G** level in *o*-dichlorobenzene (PCM). The Δq values (elementary charge in e) of the corresponding excited states and their electronic nature (LE, CT, or LE&CT) are also provided.

State	ΔE	f	Δq	Nature
S_1	3.234	0.168	0.100	LE
S_2	3.303	0.707	0.176	LE
S_3	3.408	0.676	–0.001	LE
S_4	3.422	0.490	0.017	LE
S_5	3.538	0.043	1.104	CT
S_6	3.573	0.002	0.883	LE&CT

Table S8. Vertical excitation energies (ΔE, in eV) and oscillator strengths (f) for the lowest-energy singlet excited states of the supramolecular D−A truxTTF•$C_{30}H_{12}$ complex calculated at the OT-LC-BLYP(ω = 0.16 Bohr^{-1})/6-31G** level in *o*-dichlorobenzene (PCM). The Δq values (elementary charge in e) of the corresponding excited states and their electronic nature (LE, CT, or LE&CT) are also provided.

State	ΔE	f	Δq	Nature
S_1	2.690	0.116	1.229	CT
S_2	2.790	0.035	0.464	LE
S_3	2.884	0.060	1.628	CT
S_4	2.950	0.020	1.637	CT
S_5	3.037	0.420	0.110	LE
S_6	3.066	0.558	0.130	LE

Table S9. Vertical excitation energies (ΔE, in eV) and oscillator strengths (f) for the lowest-energy singlet excited states of the supramolecular D−A truxTTF•$C_{30}H_{12}$ complex calculated at the OT-LC-ωPBE(ω = 0.15 Bohr^{-1})/6-31G** level in *o*-dichlorobenzene (PCM). The Δq values (elementary charge in e) of the corresponding excited states and their electronic nature (LE, CT, or LE&CT) are also provided.

State	ΔE	f	Δq	Nature
S_1	2.624	0.111	1.456	CT
S_2	2.734	0.021	0.308	LE
S_3	2.804	0.051	1.715	CT
S_4	2.873	0.013	1.766	CT
S_5	2.982	0.422	0.116	LE
S_6	3.012	0.593	0.172	LE

Table S10. Vertical excitation energies (ΔE, in eV) and oscillator strengths (f) for the lowest-energy singlet excited states of the supramolecular D−A truxTTF•$C_{30}H_{12}$ complex calculated at the OT-ωB97X-D(ω = 0.12 Bohr^{-1})/6-31G** level in *o*-dichlorobenzene (PCM). The Δq values (elementary charge in e) of the corresponding excited states and their electronic nature (LE, CT, or LE&CT) are also provided.

State	ΔE	f	Δq	Nature
S_1	2.872	0.124	0.731	LE&CT
S_2	2.958	0.122	0.848	LE&CT
S_3	3.062	0.109	1.555	CT
S_4	3.131	0.043	1.518	CT
S_5	3.167	0.536	0.173	LE
S_6	3.192	0.533	0.201	LE

S2.2 Low-Lying Excited States at OT-LC-BLYP($\omega = 0.16$ Bohr^{-1})

Table S11 collects the vertical excitation energies (ΔE), the oscillator strengths (f), and the Δq values calculated for the low-lying singlet excited states of the truxTTF•$C_{30}H_{12}$ heterodimer and the isolated truxTTF compound at the OT-LC-BLYP($\omega = 0.16$ Bohr^{-1})/6-31G** level in the presence of *o*-dichlorobenzene within the state-specific PCM (SS-PCM) approach.

Table S11. Vertical excitation energies (ΔE, in eV), oscillator strengths (f), and Δq values (elementary charge in e) for the supramolecular D−A truxTTF•$C_{30}H_{12}$ complex and the isolated truxTTF compound calculated at the OT-LC-BLYP($\omega = 0.16$ Bohr^{-1})/6-31G** level in *o*-dichlorobenzene within the SS-PCM approach.

truxTTF•$C_{30}H_{12}$					truxTTF		
Transitions	ΔE^a	f	Δq^b	Nature	Transitions	ΔE	f
GS → CT_1	2.22 (2.69)	0.419	1.23	CT		---	---
GS → CT_2	2.29 (2.88)	0.017	0.46	CT		---	---
GS → CT_3	2.35 (2.95)	0.001	1.63	CT		---	---
GS → LE_1	2.75 (2.79)	0.016	1.64	LE	GS → LE_1	2.76	0.0004
GS → LE_2	3.07 (3.04)	0.201	0.11	LE	GS → LE_2	3.09	0.3393
GS → LE_3	3.09 (3.07)	0.317	0.13	LE	GS → LE_3	3.09	0.3397

[a] Values within parentheses correspond to vertical excitation energies computed within the linear-response PCM approach (Table S8). [b] The Δq values provided have been evaluated within the linear-response PCM approach.

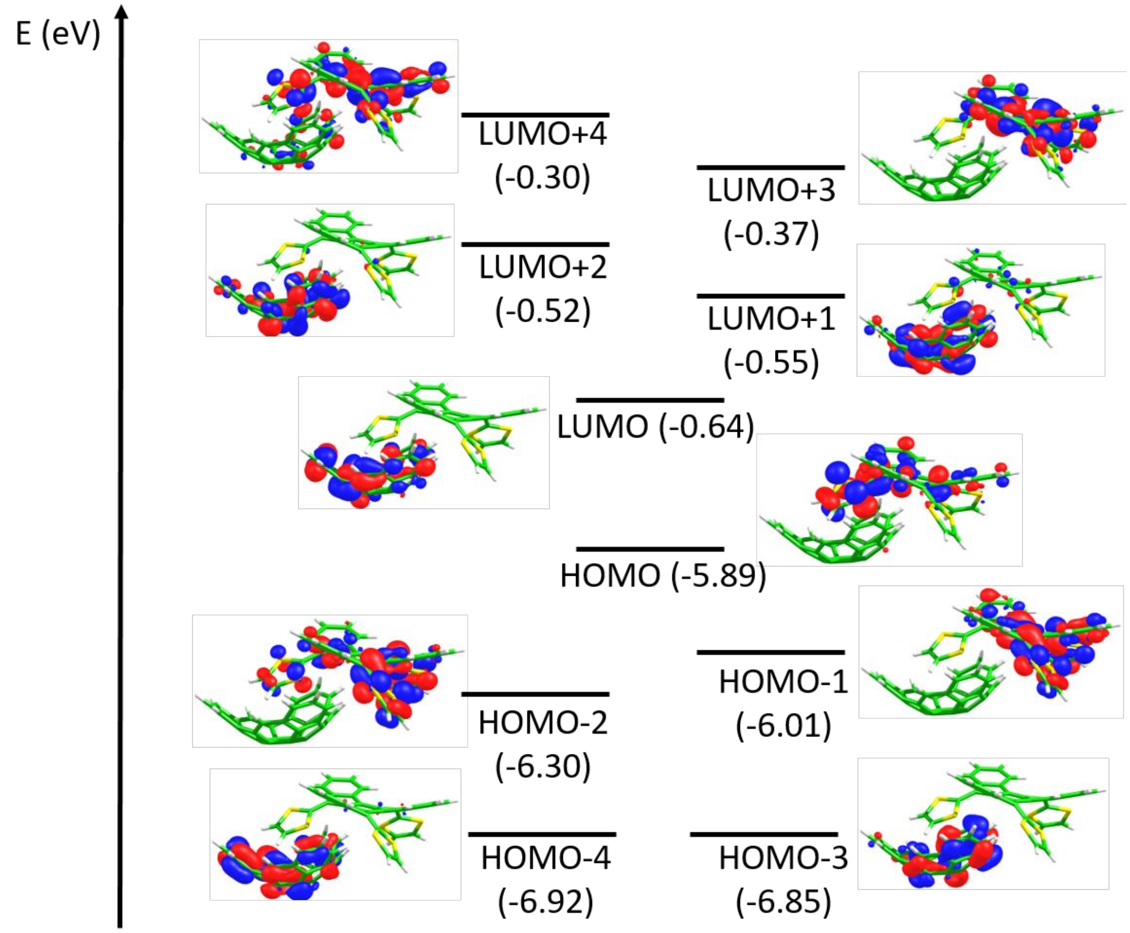

Figure S3. Diagram of the frontier molecular orbitals calculated for structure **4** of the truxTTF•$C_{30}H_{12}$ complex at the OT-LC-BLYP(ω = 0.16 Bohr^{-1})/6-31G** level in *o*-dichlorobenzene.

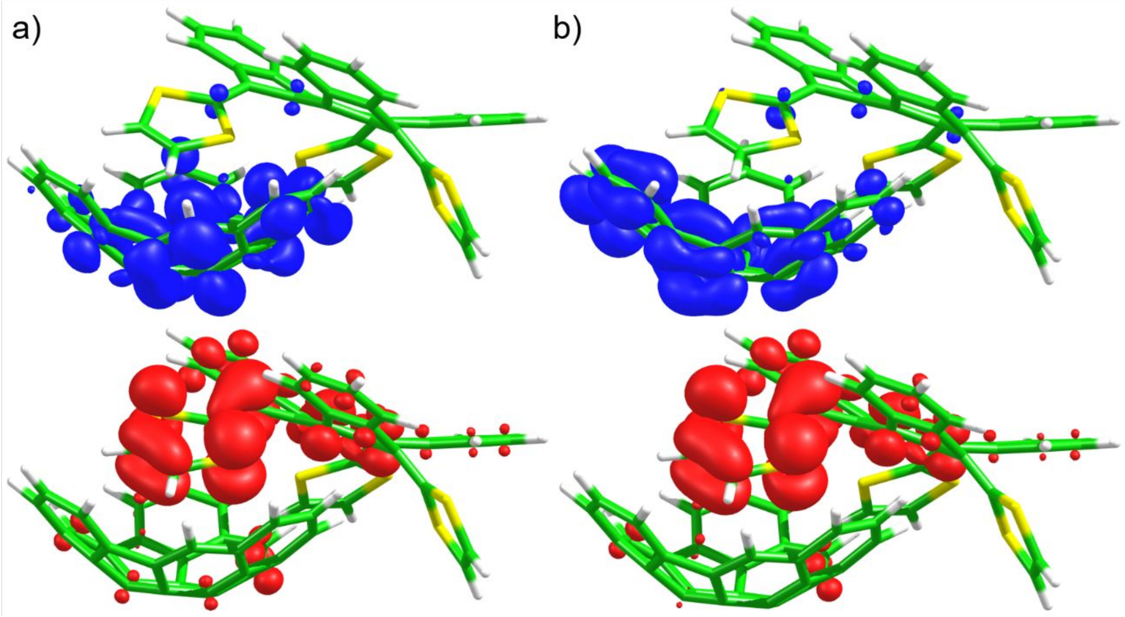

Figure S4. Attachment (top) and detachment (bottom) densities calculated for the GS → CT_2 (a) and GS → CT_3 (b) transitions of the truxTTF•$C_{30}H_{12}$ heterodimer at the OT-LC-BLYP($\omega = 0.16$ Bohr^{-1})/6-31G** level in *o*-dichlorobenzene within the PCM approach.

S3. Internal Reorganization Energy and Huang-Rhys Factors

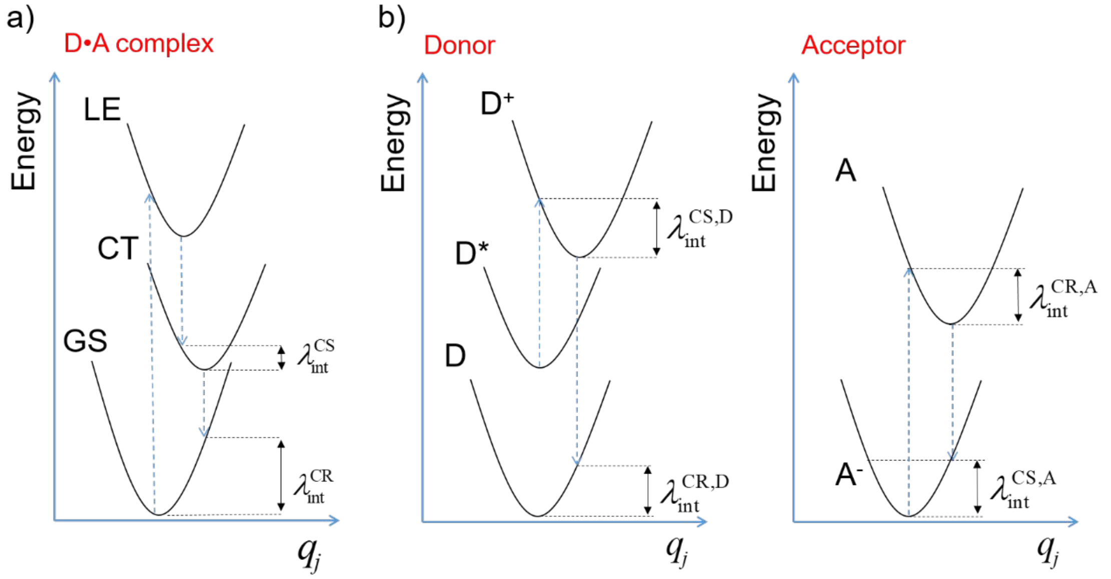

Figure S5. (a) Scheme of internal reorganization energy (λ_{int}) for a generic electron transfer process in a D−A supramolecular complex. (b) Alternative view of λ_{int} separated in the donor (D) and acceptor (A) contributions; D^+/A^- denotes the donor/acceptor in its cationic/anionic states and D* denotes the excited neutral donor unit.

Figure S5 displays a scheme of the different internal reorganization energy components for the CS and CR processes of the isolated D/A units in a generic D−A interface. For the CS process (Eq. S5), the internal reorganization energy of the donor is computed as the difference in the cation potential energy surface between the energy at the excited state minimum-energy geometry ($E_{D^+}^{D^*}$) and the energy at the optimized minimum-energy

geometry $(E_{\mathrm{D^+}}^{\mathrm{D^+}})$. In a similar way, the internal reorganization energy of the acceptor is computed as the difference in the anion potential energy surface between the energy at the neutral minimum-energy geometry $(E_{\mathrm{A^-}}^{\mathrm{A}})$ and the energy at the optimized minimum-energy geometry $(E_{\mathrm{A^-}}^{\mathrm{A^-}})$. For the CR process, the internal reorganization energy was analogously computed as the differences in the respective potential energy surfaces of the neutral donor and acceptor species between the energy at the charged (cation/anion) minimum-energy geometry $(E_{\mathrm{D}}^{\mathrm{D^+}} / E_{\mathrm{A}}^{\mathrm{A^-}})$ and the energy at the neutral equilibrium geometry (E_D^D / E_A^A).

$$\lambda_{\mathrm{int}}^{\mathrm{CS}} = \lambda_{\mathrm{int}}^{\mathrm{CS,D}} + \lambda_{\mathrm{int}}^{\mathrm{CS,A}} = (E_{\mathrm{D^+}}^{\mathrm{D^*}} - E_{\mathrm{D^+}}^{\mathrm{D^+}}) + (E_{\mathrm{A^-}}^{\mathrm{A}} - E_{\mathrm{A^-}}^{\mathrm{A^-}}) \qquad \text{Eq. S5}$$

$$\lambda_{\mathrm{int}}^{\mathrm{CR}} = \lambda_{\mathrm{int}}^{\mathrm{CR,D}} + \lambda_{\mathrm{int}}^{\mathrm{CR,A}} = (E_{\mathrm{D}}^{\mathrm{D^+}} - E_{\mathrm{D}}^{\mathrm{D}}) + (E_{\mathrm{A}}^{\mathrm{A^-}} - E_{\mathrm{A}}^{\mathrm{A}}) \qquad \text{Eq. S6}$$

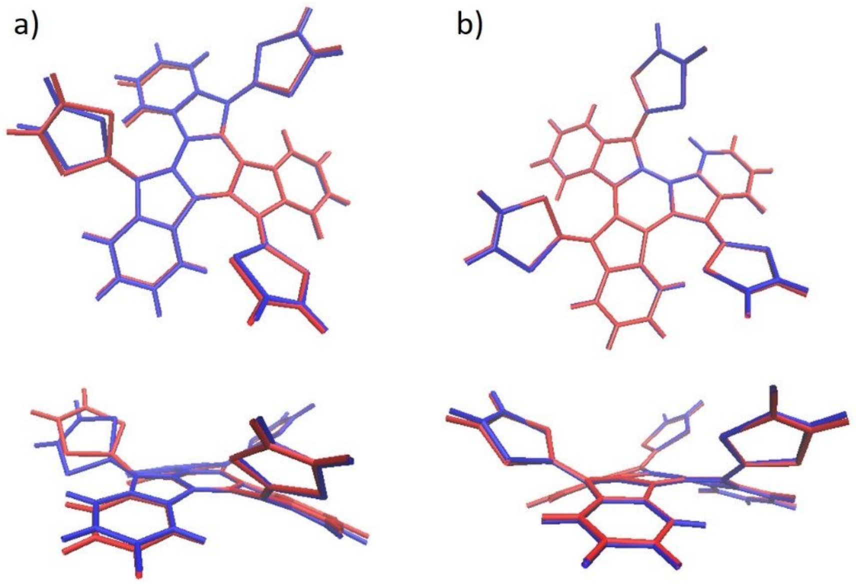

Figure S6. Top (top) and side (bottom) view of the OT-LC-BLYP-optimized structures of truxTTF used to compute the Huang−Rhys factors. (a) Superposed equilibrium geometries of the D* (blue) and D^+ (red) species used in the CS process. (b) Superposed equilibrium geometries of the D^+ (red) and D (blue) species used in the CR process.

Huang−Rhys (HR) factors were computed as explained by Malagoli *et al.*,[9] using the ionic (D^+/A^-) and neutral (D/D*/A) optimized geometries for the CS and CR events. Nevertheless, a large geometrical change in the donor truxTTF along the $D^* \rightarrow D^+$ transition has been found owing to the significant rotation of a dithiole ring (Figure S6). The dithiole rotation is responsible for an imaginary low-frequency normal mode (156*i* cm^{-1}) in the donor excited-state potential energy surface. This significant geometry deformation hinders the standard estimate of HR factors between the two D* and D^+ equilibrium geometries due to the large change of the frequencies and normal modes along the relaxation pathway. To solve this, a numerical procedure was used where

intermediate structures between the equilibrium structures from D* to D^+ were generated in such a way that small displacements between consecutive structures are guaranteed and, therefore, the standard procedure is valid and HR factors can be calculated.

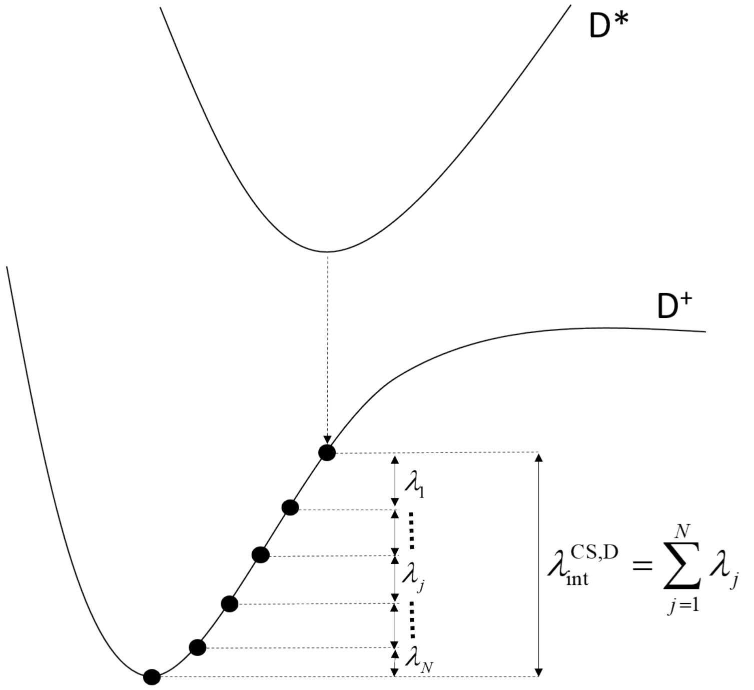

Figure S7. Scheme of the computational procedure employed to compute the Huang−Rhys factors for the D* → D^+ transition involving large displacements.

Figure S7 graphically explains the procedure followed. As stated above, ten consecutive structures were computed where the first and last structures correspond to the minimum energy geometries of the D^+ and D^* states. Then, frequencies and normal mode vectors were calculated at each geometry and HR factors were standardly computed between consecutive structures. This procedure minimized the large error obtained with the standard two-geometries estimation since frequencies at intermediate geometries are employed. Therefore, variation on the frequency wavenumber and in the normal mode vectors is taken into account. In this way, the analysis of the reorganization energy can be computed as usual for each step (*j*) and can be decomposed into the different normal

modes as $\lambda_j = \sum_k S_{k,j}\nu_{k,j}$. By summation over all the steps, we recover the reorganization energy along the complete path by $\lambda_{\mathrm{int}}^{\mathrm{CS,D}} = \sum_j^N \lambda_j$ (Figure S7). The decomposition of the reorganization energy into the different normal modes is displayed in Figure 5 (main text), where an average of the frequency of each normal mode along all the steps is used in the *x* axis according to Eq. S7.

$$\nu_k = \frac{\sum_j^N S_{k,j}\nu_{k,j}}{\sum_j^N S_{k,j}} \qquad \text{Eq. S7}$$

S4. External Reorganization Energy

The external reorganization energy for the CS and CR processes is evaluated as the difference between the total reorganization energy (λ) calculated with the "Equilibrium-vs-NonEquilibrium" state-specific PCM approach and the internal $\lambda_{\mathrm{int}}^{\mathrm{CS/CR}}$ contribution (previously discussed) for the isolated monomers (Eqs. S5 and S6).

$$\lambda_{\mathrm{ext}}^{\mathrm{CS/CR}} = \lambda^{\mathrm{CS/CR}} - \lambda_{\mathrm{int}}^{\mathrm{CS/CR}} \qquad \text{Eq. S8}$$

λ of the donor in CS and CR processes is calculated according to Eqs. S9 and S10. Then, the external reorganization energy is computed as the difference between the total λ and the internal reorganization energy (Eq. S8). An analogous procedure is adopted for the acceptor using Eqs. S11 and S12. Energies in Eqs. S9–S12 correspond to the energy of the donor/acceptor, indicated as the subscript, at the minimum-energy geometry of the species indicated in the superscript. *NonEq* and *Eq* superscripts denote the NonEquilibrium or Equilibrium state-specific solvent interaction. For instance, $E_{D^+}^{D^*,NonEq}$ denotes the energy of D^+ at the excited state (D^*) minimum-energy geometry with the NonEquilibrium approach, which implies that only the fast polarization modes of the solvent are adapted to the D^+ state, whereas the slow modes are frozen as in the D^* state. In contrast, $E_{D^+}^{D^+,Eq}$ means the energy of D^+ in its minimum-energy geometry with the solvent totally relaxed (Equilibrium).

$$\lambda_D^{CS} = E_{D^+}^{D^*,NonEq} - E_{D^+}^{D^+,Eq} \qquad \text{Eq. S9}$$

$$\lambda_D^{CR} = E_D^{D^+,NonEq} - E_D^{D,Eq} \qquad \text{Eq. S10}$$

$$\lambda_A^{CS} = E_{A^-}^{A,NonEq} - E_{A^-}^{A^-,Eq} \qquad \text{Eq. S11}$$

$$\lambda_A^{CR} = E_A^{A^-,NonEq} - E_A^{A,Eq} \qquad \text{Eq. S12}$$

S5. Analysis of truxTTF•$C_{30}H_{12}$ Supramolecular Structures

Table S12. Relevant parameters (V_{ij}, ΔG_{ij}, λ_c, S_{eff}, and ν_{eff}, in eV) and k_{CS} and k_{CR} rate constants (in s^{-1}) calculated for the different electron-transfer pathways in structure **2** of the truxTTF•$C_{30}H_{12}$ heterodimer.

Transition	$V_{ij}{}^{a}$	$-\Delta G_{ij}$	λ_c	S_{eff}	$\nu_{eff}{}^{b}$	k_{ij}
CS process						
$LE_1 \rightarrow CT_1$	0.003	0.66	1.23	2.66	0.085 (683)	4.7×10^9
$LE_1 \rightarrow CT_2$	0.000 (10^{-5})	0.21	1.23	2.66	0.085 (683)	1.2×10^2
$LE_1 \rightarrow CT_3$	0.052	0.21	1.23	2.66	0.085 (683)	3.2×10^9
$LE_2 \rightarrow CT_1$	0.012	0.74	1.23	2.66	0.085 (683)	1.4×10^{11}
$LE_2 \rightarrow CT_2$	0.009	0.29	1.23	2.66	0.085 (683)	4.0×10^8
$LE_2 \rightarrow CT_3$	0.000 (10^{-4})	0.29	1.23	2.66	0.085 (683)	2.6×10^5
$LE_3 \rightarrow CT_1$	0.008	0.75	1.23	2.66	0.085 (683)	6.9×10^{10}
$LE_3 \rightarrow CT_2$	0.021	0.30	1.23	2.66	0.085 (683)	2.3×10^9
$LE_3 \rightarrow CT_3$	0.000 (10^{-4})	0.30	1.23	2.66	0.085 (683)	6.1×10^5
CR process						
$CT_1 \rightarrow GS$	0.027	1.85	0.75	0.97	0.108 (872)	4.3×10^9
$CT_2 \rightarrow GS$	0.009	2.30	0.75	0.97	0.108 (872)	1.0×10^5
$CT_3 \rightarrow GS$	0.013	2.30	0.75	0.97	0.108 (872)	2.1×10^5

[a] Values within parentheses correspond to the order of magnitude of V_{ij} lower than 1 meV. [b] Values within parentheses are in cm^{-1}.

Table S13. Relevant parameters (V_{ij}, ΔG_{ij}, λ_c, S_{eff}, and ν_{eff}, in eV) and k_{CS} and k_{CR} rate constants (in s^{-1}) calculated for the different electron-transfer pathways in structure **3** of the truxTTF•$C_{30}H_{12}$ heterodimer.

Transition	V_{ij}	$-\Delta G_{ij}$	λ_c	S_{eff}	$\nu_{eff}{}^{a}$	k_{ij}

CS process						
$LE_1 \rightarrow CT_1$	0.022	0.34	1.23	2.66	0.085 (683)	4.3×10^9
$LE_2 \rightarrow CT_1$	0.049	0.44	1.23	2.66	0.085 (683)	8.6×10^{10}
$LE_3 \rightarrow CT_1$	0.017	0.45	1.23	2.66	0.085 (683)	1.2×10^{10}
CR process						
$CT_1 \rightarrow GS$	0.028	2.02	0.75	0.97	0.108 (872)	1.7×10^8

[a] Values within parentheses are in cm^{-1}.

Table S14. Adiabatic energy differences (ΔE) between the ground state and the LE and CT excited states calculated at the OT-LC-BLYP/6-31G** level in *o*-dichlorobencene (SS-PCM) for structures **1**–**4** of the truxTTF•$C_{30}H_{12}$ heterodimer by following the protocol specified in the main text.

	Structure **1**[a]		Structure **2**[a]		Structure **3**[a]		Structure **4**[a]	
State	ΔE^b	Nature	ΔE	Nature	ΔE	Nature	ΔE	Nature
S_1	2.85	LE	1.85	CT	2.02	CT	1.75	CT
S_2	3.05	LE	2.30	CT	2.34	LE	2.02	CT
S_3	3.06	LE	2.30	CT	2.46	LE	2.08	CT
S_4	3.08	LE	2.51	LE	2.47	LE	2.49	LE
S_5	3.28	LE&CT	2.59	LE			2.58	LE
S_6	3.35	LE&CT	2.60	LE			2.60	LE

[a] Optimally tuned ω values of 0.18, 0.17, 0.14 and 0.16 $Bohr^{-1}$ were used for structures **1**, **2**, **3** and **4**, respectively. [b] Energies given for structure **1** were computed using the linear-response PCM.

Rates constants for the most relevant $LE_1 \rightarrow CT_1$, $LE_3 \rightarrow CT_1$ and $CT_1 \rightarrow GS$ pathways were also computed by using the popular Marcus theory for comparison purposes. The rate values obtained (8.6×10^{11}, 1.4×10^{12} s^{-1} and 2.0×10^{8} s^{-1}, respectively) are smaller than those calculated with the MLJ expression due to the lack of quantum tunneling effects.

S6. Kinetic Model

To take into account all possible deactivation pathways in a global manner, a kinetic model based on a Pauli master equation is proposed. The master equation is written as follows:

$$\frac{\partial \rho_i}{\partial t} = \sum_{j \neq i} \left(k_{j \to i} \rho_j - k_{i \to j} \rho_i \right), \qquad \text{Eq. S13}$$

where ρ_i corresponds to the population of the electronic state *i*. $k_{i \to j}$ is the rate constant calculated for the $i \to j$ electronic transition. The CS and CR rates introduced in the kinetic model are gathered in Tables S12 and S13 for structures **2** and **3**, respectively, and in Table 3 in the main text for structure **4**. The internal conversion rates between states of the same nature were tentatively set according to values reported in the literature (1.0 × 10^{13} s^{-1} for transitions (forward and backward) between quasi-degenerate LE_2/LE_3 and CT_2/CT_3 pairs).[10,11] For downhill internal conversions (*e.g.*, $LE_2 \rightarrow LE_1$, $LE_3 \rightarrow LE_1$, $CT_2 \rightarrow CT_1$, and $CT_3 \rightarrow CT_1$) a rate constant of 2.0 × 10^{11} s^{-1} was employed whereas for the opposite uphill transitions a rate ten times slower (2.0 × 10^{10} s^{-1}) was set. These rates are reasonable for transition between states with small energy gaps $\left(\Delta E \approx 0.2 - 0.4 \text{ eV}\right)$. Finally, the master equation (Eq. S13) was solved by numerical integration by using the stiff backward differentiation formula method as implemented in the GNU Octave

software. The total simulation time was established to be 100 ns with a time step of 10 fs. All the initial populations (ρ_i) for all electronic states were set to be 0 but for the bright local states (LE_2 and LE_3) for which initial values of 0.5 were assumed.

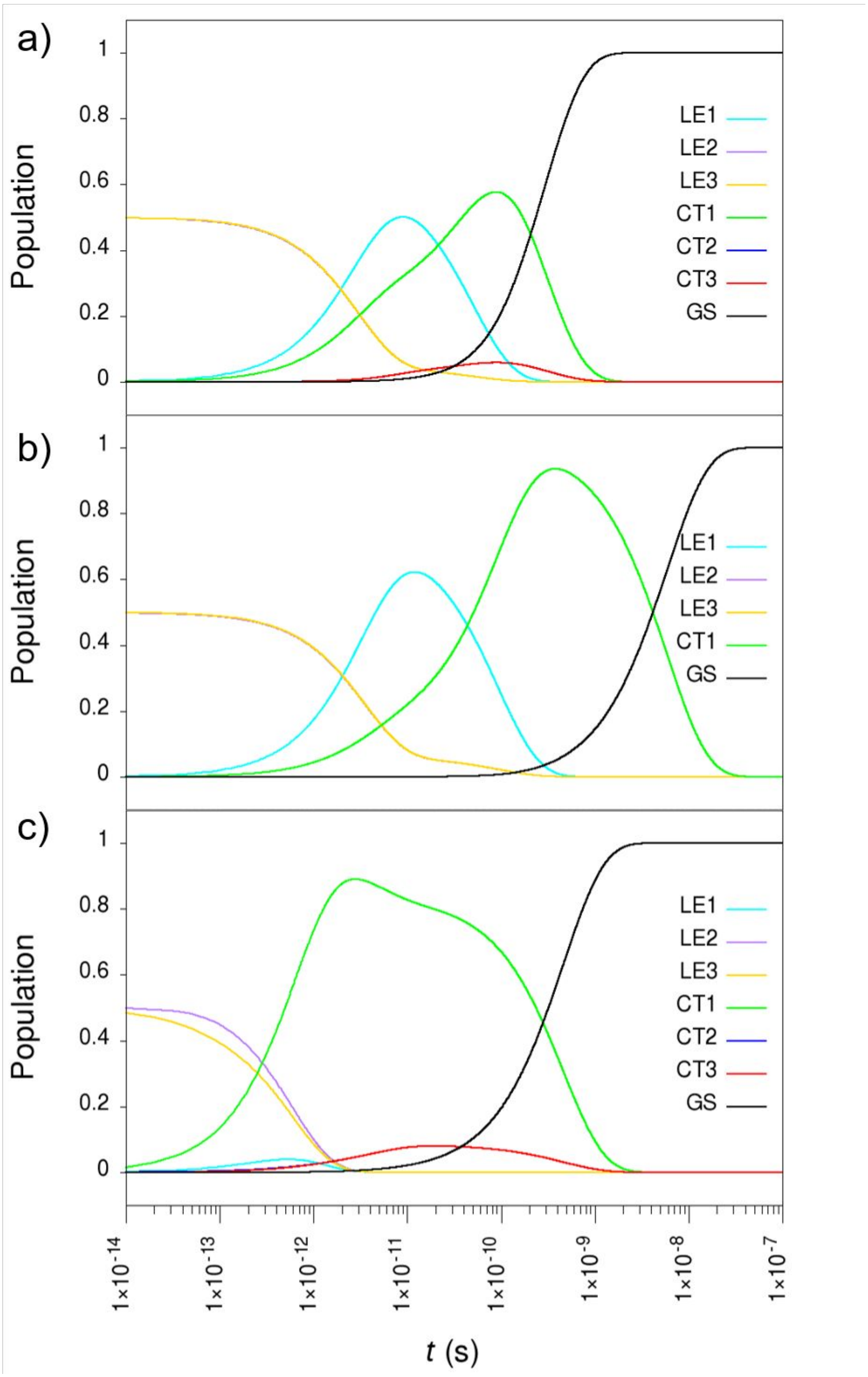

Figure S8. Time evolution of the population of each electronic state calculated for structures **2** (a), **3** (b) and **4** (c) according to the kinetic model described above. Time (*x* axis) is represented in logarithmic scale.

S7. References

(1) Cave, R. J.; Newton, M. D. *Chem. Phys. Lett.* **1996**, *249*, 15–19.

(2) Yang, C.-H.; Hsu, C.-P. *J. Chem. Phys.* **2013**, *139*, 154104.

(3) Stein, T.; Kronik, L.; Baer, R. *J. Am. Chem. Soc.* **2009**, *131*, 2818–2820.

(4) Gallego, M.; Calbo, J.; Aragó, J.; Krick Calderon, R. M.; Liquido, F. H.; Iwamoto, T.; Greene, A. K.; Jackson, E. A.; Pérez, E. M.; Ortí, E.; Guldi, D. M.; Scott, L. T.; Martín, N. *Angew. Chem. Int. Ed.* **2014**, *53*, 2170–2175.

(5) Tsuneda, T.; Hirao, K. *Wiley Interdiscip. Rev. Comput. Mol. Sci.* **2014**, *4*, 375–390.

(6) Vydrov, O. A.; Scuseria, G. E. *J. Chem. Phys.* **2006**, *125*, 234109.

(7) Gräfenstein, J.; Cremer, D. *Theor. Chem. Acc.* **2009**, *123*, 171–182.

(8) Lundberg, M.; Siegbahn, P. E. M. *J. Chem. Phys.* **2005**, *122*, 224103.

(9) Malagoli, M.; Coropceanu, V.; da Silva Filho, D. A.; Brédas, J. L. *J. Chem. Phys.* **2004**, *120*, 7490–7496.

(10) Turro, N. J. *Modern Molecular Photochemistry*; University Science Books,U.S.,

1991.

(11) Klessinger, M.; Michl, J. *Excited States and Photochemistry of Organic Molecules*; Wiley-VCH Verlag GmbH, 1995.
